# Symmetry-based pulsed DNP and EPR sequences

**DOI:** 10.1126/sciadv.aef4949

**Published:** 2026-09-16

**Authors:** Zhenfeng Pang, Giuseppe Sicoli, Benoit Driesschaert, Jean-Paul Amoureux, Hervé Vezin, Kong Ooi Tan

**Affiliations:** ^1^ Chimie Physique et Chimie du Vivant (CPCV), UMR 8228, Département de Chimie, École Normale Supérieure, PSL Université, Sorbonne Université, CNRS, 75005 Paris, France.; ^2^ University of Lille, CNRS UMR 8516−LASIRE−Laboratoire de Spectroscopie pour les Interactions la Réactivité et l’Environnement, Lille 59000, France.; ^3^ Department of Pharmaceutical Sciences, School of Pharmacy, West Virginia University, Morgantown, WV 26506, USA.; ^4^ Univ. Lille, CNRS, Centrale Lille, Univ. Artois, UMR 8181, UCCS–Unité de Catalyse et Chimie du Solide, F-59000 Lille, France.

## Abstract

Pulsed microwave irradiation provides more versatile control over spin dynamics than continuous-wave (CW) methods. This study adapts symmetry-based dipolar recoupling sequences, which are originally designed for magic angle spinning (MAS) NMR, to exploit electron-nuclear interactions for dynamic nuclear polarization (DNP) and pulsed electron paramagnetic resonance (EPR). Using the trityl radical OX071 at X-band (0.35 tesla), phase-modulated microwave pulses were synchronized with the nuclear Larmor period. Experimental results show effective DNP performance and electron-nuclear distance measurements in EPR, consistent with numerical simulations and theory. This work shows that many MAS NMR pulse sequences can be adapted to static pulsed EPR and DNP with minor modifications. This cross-disciplinary approach provides a versatile platform for designing optimized, application-specific magnetic resonance methodologies across various field strengths, subject to instrumental capabilities.

## INTRODUCTION

Magnetic resonance is a powerful characterization tool for probing systems ranging from small molecules and inorganic materials to biological macromolecules. A defining strength of magnetic resonance is the exquisite control over spin dynamics afforded by a vast library of pulse sequences. In solid-state nuclear magnetic resonance (NMR), hundreds of sequences exist to measure chemical-shift tensors, dipolar couplings, and relaxation parameters (*1*–*3*). The selection of an optimal sequence is a strategic process dictated by experimental conditions such as spin species, molecular moieties, maximal radio frequency (RF), and static magnetic field strengths. To address increasingly complex chemical and biological problems, it is crucial to continue expanding this library with sequences that offer enhanced performance and versatility.

Although NMR, electron paramagnetic resonance (EPR), and DNP share the same fundamental principles, many pulse sequences remain confined within their respective subdomains. For example, magic angle spinning (MAS) NMR uses a time-dependent Hamiltonian that has necessitated an advanced theoretical framework seldom applied to static samples in EPR (*4*–*7*). Recently, however, it has been shown that concepts can be unified across these seemingly distinct techniques using a common mathematical framework (*8*–*10*). A notable example is PulsePol (*11*), a pulsed DNP technique for nitrogen-vacancy (NV) centers, where the microwave-induced spin dynamics were found to be mathematically analogous to symmetry-based recoupling in MAS NMR (*12*). This conceptual bridge has since been extended to singlet-triplet conversion in solution-state NMR and the adoption of MAS-inspired sequences (e.g., TOP-DNP, XiX, TPPM, BEAM-DNP, etc.) for pulsed DNP (*13*–*17*). These developments suggest that the mathematical formalisms describing spin dynamics can be adapted across EPR, DNP, and NMR.

Building on these shared mathematical principles, we demonstrate that the family of symmetry-based $CN_{n}^{\nu }$ and $RN_{n}^{\nu }$ pulse sequences (*18*–*20*), which are well established in MAS NMR, can be translated to static pulsed DNP and EPR. These sequences offer more than just high performances; and their expansive combinatorial space (defined by the integers *N*, *n*, and ν as well as basic element types and various supercycling strategies) (*21*, *22*) provides the flexibility to tune interactions for specific needs. While the fundamental theory of symmetry-based sequences under MAS is well documented in the NMR literature (*18*), this work focuses on their adaptation to static samples in the context of DNP and EPR. We show that this transformation provides a systematic approach to designing optimized, application-specific methodologies that substantially enrich the existing pulse library for magnetic resonance at various fields.

## RESULTS AND DISCUSSION

### Theory of symmetry-based pulsed DNP

The Hamiltonian of a two-spin electron (*S*)–nucleus (*I*) system in the electron-rotating frame is

$$\hat{H}=-\omega_{0I}{\hat{I}}_{z}+A_{\text{zz}}{\hat{S}}_{z}{\hat{I}}_{z}+A_{\text{zx}}{\hat{S}}_{z}{\hat{I}}_{x}+A_{\text{zy}}{\hat{S}}_{z}{\hat{I}}_{y}$$ (1)

where $A_{\text{zz}}$ and $A_{\text{zx}\left(y\right)}$ are the secular and pseudo-secular components of the hyperfine interaction, respectively, and $\omega_{0I}$ is the nuclear Larmor frequency. The hyperfine interaction can be rewritten using irreducible spherical tensor operators (see the Supplementary Materials)

$$\hat{H}=-\omega_{0I}{\hat{I}}_{z}+\sum_{m=-1}^{1}{\left(\;-\;1\right)}^{m}\omega_{m}{\hat{T}}_{1,-m}^{\left(I\right)}{\hat{T}}_{1,0}^{\left(S\right)}$$ (2)

where ${\hat{T}}_{1,0}^{\left(I\right)}={\hat{I}}_{z}$, ${\hat{T}}_{1,\pm 1}^{\left(I\right)}=\mp \;\frac{1}{\sqrt{2}}\left({\hat{I}}_{x}\pm i{\hat{I}}_{y}\right)$, ${\hat{T}}_{1,0}^{\left(S\right)}={\hat{S}}_{z}$, ${\hat{T}}_{1,\pm 1}^{\left(S\right)}=\mp \;\frac{1}{\sqrt{2}}$$\left({\hat{S}}_{x}\;\pm \;i{\hat{S}}_{y}\right)$, $\omega_{0}=A_{\text{zz}}$, and $\omega_{\pm 1}=\mp \frac{1}{\sqrt{2}}\left(A_{\text{zx}}\;\pm \;iA_{\text{zy}}\right)$. Then, an interaction-frame transformation is performed using the nuclear Zeeman propagator, ${\hat{U}}_{1}\left(t\right)=\text{exp}\left(i\omega_{0I}t{\hat{I}}_{z}\right)$, to obtain a time-dependent Hamiltonian in the electron-nucleus double rotating frame

$$\begin{array}{c} {\hat{U}}_{1}^{-1}\left(t\right)\hat{H}{\hat{U}}_{1}\left(t\right)\;+\;\omega_{0I}{\hat{I}}_{z}=\sum_{m=-1}^{1}{\left(\;-\;1\right)}^{m}\omega_{m}\text{exp}\left(-i\omega_{0I}t{\hat{I}}_{z}\right){\hat{T}}_{1,-m}^{\left(I\right)}\text{exp}\left(i\omega_{0I}t{\hat{I}}_{z}\right){\hat{T}}_{1,0}^{\left(S\right)}\\ =\sum_{m=-1}^{1}\omega_{\text{hf}}^{-m}\text{exp}\left(\mathrm{im}\omega_{0I}t\right){\hat{T}}_{1,-m}^{\left(I\right)}{\hat{T}}_{1,0}^{\left(S\right)}\\ =\sum_{m^{\prime }=-1}^{1}\omega_{\text{hf}}^{m^{\prime }}\text{exp}\left(-im^{\prime }\omega_{0I}t\right){\hat{T}}_{1,m^{\prime }}^{\left(I\right)}{\hat{T}}_{1,0}^{\left(S\right)}\;\left(\text{with}\;m^{\prime }=-m\right)\\ =\sum_{m=-1}^{1}\omega_{\text{hf}}^{m}\text{exp}\left(-\mathrm{im}\omega_{0I}t\right){\hat{T}}_{1,m}^{\left(I\right)}{\hat{T}}_{1,0}^{\left(S\right)}\;\left(\text{changing}\;m^{\prime }\;\text{to}\;m\right) \end{array}$$ (3)

where $\omega_{\text{hf}}^{m}={\left(\;-\;1\right)}^{m}\omega_{-m}$. This expression is mathematically analogous to the Hamiltonian of a solid-state NMR system under MAS before RF irradiation

$$\begin{array}{c} {\hat{H}}_{\text{int},\;\text{MAS}}\left(t\right)=\sum_{\Lambda ,l,m,\lambda }\omega_{\mathrm{lm}}^{\Lambda }\text{exp}\left(\mathrm{im}\omega_{r}t\right){\hat{T}}_{\lambda 0}^{\Lambda }\\ =\sum_{m=-2}^{2}\omega_{D}^{m}\text{exp}\left(\mathrm{im}\omega_{r}t\right){\hat{T}}_{1,0}^{\left(I\right)}{\hat{T}}_{1,0}^{\left(S\right)}\left(\text{by}\;\text{only considering}\;D_{\text{IS}}\right) \end{array}$$ (4)

where $\omega_{\mathrm{lm}}^{\Lambda }$ describes the strength of different types (Λ) of spin interactions, $\omega_{r}$ is the MAS spinning frequency, and ${\hat{T}}_{\lambda 0}^{\Lambda }$ is the element of rank λ spin-tensor operator. In the second line of Eq. 4, we consider only the heteronuclear dipolar coupling interaction ($D_{\text{IS}}$) with a spatial component $\omega_{D}^{m}$ because it has the closest analogy in DNP or electron-nuclear modulation experiment in EPR. By comparing the last lines in Eqs. 3 and 4, we observe that both equations are mathematically similar by inspecting (i) the time-dependent component: $\text{exp}\left(\;-\mathrm{im}\omega_{0I}t\right)\equiv \text{exp}\left(\mathrm{im}\omega_{r}t\right)$, (ii) the combined spatial and *I*-spin term: $\omega_{\text{hf}}^{m}{\hat{T}}_{1,m}^{\left(I\right)}\equiv \omega_{D}^{m}{\hat{T}}_{1,0}^{\left(I\right)}$, and (iii) the remaining (identical) *S*-spin term: ${\hat{T}}_{1,0}^{\left(S\right)}$. This similarity suggests that the recoupling techniques via rotor-synchronized pulses in MAS NMR can be adapted to electron-nucleus system if the μw pulses are synchronized with the nuclear Larmor period instead of the rotor period.

Under μw irradiation, the *S* spins undergo an interaction-frame transformation with the propagator ${\hat{U}}_{\mathrm{\mu w}}=\text{exp}\left[-i\alpha \left(t\right){\hat{S}}_{z}\right]\text{exp}\left[-i\beta \left(t\right){\hat{S}}_{y}\right]\text{exp}\left[-i\gamma \left(t\right){\hat{S}}_{z}\right]$ (*19*)

$${\hat{U}}_{\mathrm{\mu w}}^{-1}\left(t\right){\hat{T}}_{1,0}^{\left(S\right)}{\hat{U}}_{\mathrm{\mu w}}\left(t\right)\;=\sum_{\mu =-1}^{1}d_{\mu 0}^{1}\left[-\beta \left(t\right)\right]\text{exp}\left[i\text{μγ}\left(t\right)\right]{\hat{T}}_{1,\mu }^{\left(S\right)}$$ (5)

where $d_{\mu 0}^{1}\left[-\beta \left(t\right)\right]$ is the reduced Wigner matrix and $\left[\alpha \left(t\right),\beta \left(t\right),\gamma \left(t\right)\right]$ are the time-dependent Euler angles encoding the μw pulses (phases, amplitudes, and widths) that induce the spin rotations, a generic expression that can be used to describe essentially almost all pulse sequences. By combining Eqs. 3 and 5, one obtains a time-dependent Hamiltonian $\hat{\tilde{H}}\left(t\right)$ in the interaction frame

$$\hat{\tilde{H}}\left(t\right)=\sum_{m=-1}^{1}\sum_{\mu =-1}^{1}\omega_{\text{hf}}^{m}d_{\mu 0}^{1}\left[-\beta \left(t\right)\right]\text{exp}\left(-\mathrm{im}\omega_{0I}t\right)\text{exp}\left[i\text{μγ}\left(t\right)\right]{\hat{T}}_{1,m}^{\left(I\right)}{\hat{T}}_{1,\mu }^{\left(S\right)}$$ (6)

**Fig. 1.**
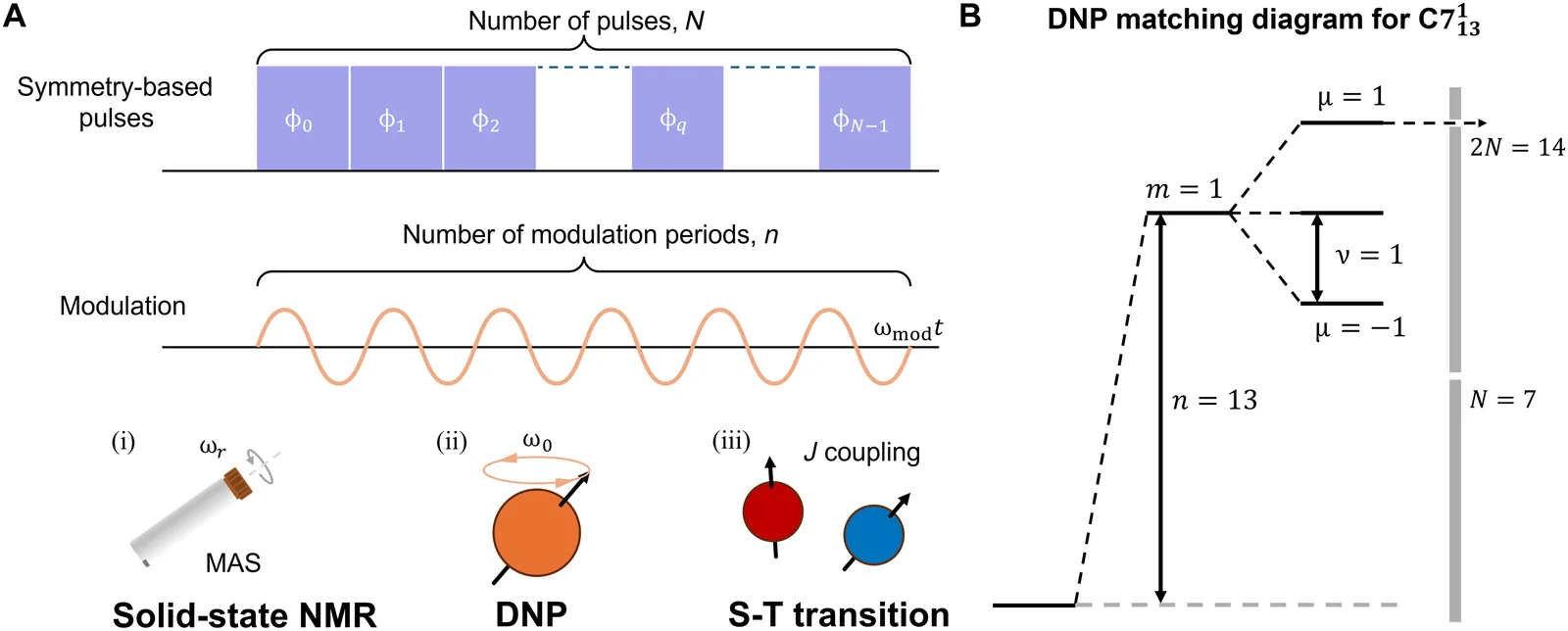
Schematic diagram of symmetry-based $CN_{n}^{\nu }$ sequences. (**A**) The sequence is comprised of *N* pulses with incrementing phases $\phi_{q}$, with a total modulation period (*N* × duration of each pulse) synchronized with either (i) *n* rotor periods in MAS NMR, (ii) *n* nuclear Larmor periods in DNP, or (iii) *n J* evolution period for singlet-triplet conversion. (**B**) The DNP matching diagram for $C7_{13}^{1}$, which elucidates that the matching condition mn+μν=zN (Eq. 9), is allowed for m=1,n=13,μ=1,ν=1,N=7, and *z* = 2.

We follow a similar analytical treatment of the MAS NMR symmetry–based sequences (*19*), where the Euler angle $\left[\alpha \left(t_{q}\right),\beta \left(t_{q}\right),\gamma \left(t_{q}\right)\right]$ accumulate as the time increments across each of the elements (or the index *q* iterates from 0 to N−1) in the symmetry-based pulses (Fig. 1). We can write the Hamiltonians ${\hat{\tilde{H}}}_{C}\left(t_{q}\right)$ and ${\hat{\tilde{H}}}_{R}\left(t_{q}\right)$ at time $t_{q}=t+q\tau$ for the $CN_{n}^{\nu }$ and $RN_{n}^{\nu }$ sequences, respectively, where 0<t< $\tau =\frac{2n\pi }{{N\omega }_{0I}}$, the duration of each element

$$\begin{array}{c} {\hat{\tilde{H}}}_{C}\left(t_{q}\right)=\sum_{\mu ,m=-1}^{1}{\hat{\tilde{H}}}_{\mu m}^{0}\left(t\right)\text{exp}\left[-i\frac{2\pi q}{N}\left(mn+\mu \nu \right)\right]\\ {\hat{\tilde{H}}}_{R}\left(t_{q}\right)=\sum_{\mu ,m=-1}^{1}\text{exp}\left(-i\frac{\text{πν}}{N}\right){\hat{\tilde{H}}}_{\mu m}^{0}\left(t\right)\text{exp}\left[-i\frac{2\pi q}{N}\left(mn+\mu \nu -\frac{N}{2}\right)\right] \end{array}$$ (7)

where

$${\hat{\tilde{H}}}_{\mu m}^{0}\left(t\right)\;=\;\omega_{\text{hf}}^{m}d_{\mu 0}^{1}\left[-\beta \left(t\right)\right]\text{exp}\left[-im\omega_{0I}t+i\mu \gamma \left(t\right)\right]{\hat{T}}_{1,m}^{\left(I\right)}{\hat{T}}_{1,\mu }^{\left(S\right)}$$ (8)

is the Hamiltonian at time t in the basic element. Following that, a nonzero time-independent effective Hamiltonian can be obtained using the average Hamiltonian theory if the following conditions are satisfied

mn+μν=zN,forCNnνmn+μν=(2z+1)N2,forRNnν (9)

where *z* is any integer. Note that the general matching condition of $RN_{n}^{\nu }$ sequences for the e-^1^H case is given by mn+μν=(2z+λ)N2, where λ = 1 is chosen for DNP to account for the rank 1 nature of the electron spin tensor upon microwave irradiation. By strategically choosing *N*, *n*, and ν, we can obtain either a ZQ [zero quantum; ${\hat{T}}_{1,\pm 1}^{\left(I\right)}{\hat{T}}_{1,\mp 1}^{\left(S\right)}$] or DQ [double quantum; ${\hat{T}}_{1,\pm 1}^{\left(I\right)}{\hat{T}}_{1,\pm 1}^{\left(S\right)}$] Hamiltonian to drive the polarization transfer (see Table 1).

**Table 1.** DNP matching condition using symmetry-based pulses (*m* = ± 1 and μ = ± 1).

	Matching condition	DQ DNP (positive)	ZQ DNP (negative)	Examples
$CN_{n}^{\nu }$	mn+μν=zN	n+ν=zN	n−ν=zN	$C7_{6}^{1}\;\left(\text{DQ}\right),C7_{6}^{-1}\left(\text{ZQ}\right)$
$RN_{n}^{\nu }$	mn+μν=(2z+1)N2	n+ν=(2z+1)N2	n−ν=(2z+1)N2	$R6_{4}^{-1}\left(\text{DQ}\right),R4_{3}^{1}\left(\text{ZQ}\right)$

Following the matching conditions given in Eq. 9, one obtains the first-order average Hamiltonian if the nuclear Larmor frequency and the electron Rabi field are larger than the hyperfine interaction

$$\begin{array}{c} \frac{f}{\tau }\int_{0}^{\tau }\mathrm{dt}{\hat{\tilde{H}}}_{\mu m}^{0}\left(t\right)=\kappa_{1m1\mu }\left(N,n,\nu \right)\omega_{\text{hf}}^{m}{\hat{T}}_{1,m}^{\left(I\right)}{\hat{T}}_{1,\mu }^{\left(S\right)}\;\\ \kappa_{1m1\mu }\left(N,n,\nu \right)=\;\frac{f}{\tau }\int_{0}^{\tau }\mathrm{dt}d_{\mu 0}^{1}\left[-\beta \left(t\right)\right]\text{exp}\left[-im\omega_{0I}t+i\text{μγ}\left(t\right)\right] \end{array}$$ (10)

where *f* is a numerical prefactor that takes the value *f* = 1 for $CN_{n}^{\nu }$ sequences and *f* = $\;\text{exp}\left(\;-\;i\frac{\text{πν}}{N}\right)\;$ for $RN_{n}^{\nu }$ sequences. The coefficient $\kappa_{1m1\mu }\left(N,n,\nu \right)$ is the scaling factor determining the strength of the “recoupled” dipolar interaction, which dictates the robustness of the pulse sequence against experimental imperfections such as field inhomogeneity, bandwidth, etc. Note that these imperfections can also affect the effective fields and consequently shift the matching conditions away from their theoretically predicted values. A primary advantage of the symmetry-based framework is that the scaling factor can be easily “tuned” or customized for specific applications by adjusting the *N*, *n*, and ν integers and by the choice of the $CN_{n}^{\nu }$ or $RN_{n}^{\nu }$ element (corresponding to effective 2π or π rotation, respectively), which lead, in principle, to an infinite number of combinations.

### Experimental results of symmetry-based DNP

**Fig. 2.**
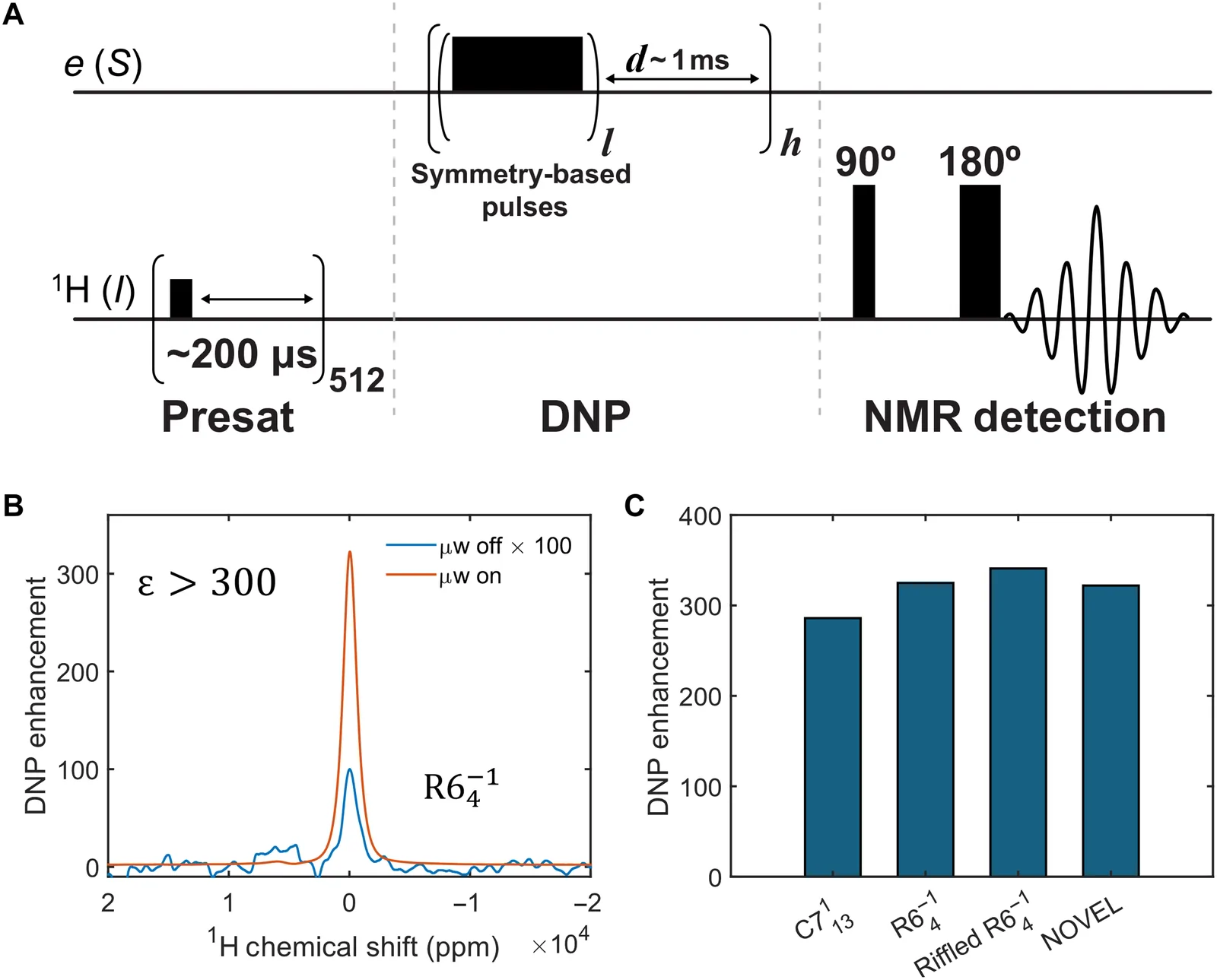
Experimental results of symmetry-based pulsed DNP. (**A**) Pulse sequence used to observe DNP signal. (**B**) NMR spectra of 5 mM OX071 with symmetry-based pulsed DNP using $R6_{4}^{-1}$ with microwave on/off. (**C**) Comparison of DNP enhancement of nuclear orientation via electron spin locking (NOVEL) (*36*) and symmetry-based pulsed DNP methods including $C7_{13}^{1}$, $R6_{4}^{-1}$, and riffled $R6_{4}^{-1}$ (Fig. 3A). ppm, parts per million.

To verify the adaptability of symmetry-based sequences for DNP, experiments were performed on 5 mM OX071 in a “DNP juice” matrix (*d*_8_-glycerol/D_2_O/H_2_O, 6:3:1, v/v/v) at 80 K and 0.35 T. The experimental protocol (Fig. 2A) began with NMR presaturation pulses, followed by a DNP block consisting of a symmetry-based sequence repeated *l* times on the *S* channel. A delay *d ∼* 1 ms was inserted between blocks to allow ${\hat{S}}_{z}$ recovery via *T*_1e_ relaxation. The entire cycle was repeated *h* times before NMR acquisition using a spin echo. The parameters *l* and *h* were optimized experimentally for each sequence (Table 2 and fig. S1).

**Table 2.** Optimized experimental parameters for symmetry-based pulsed DNP.

	*l*	Basic element	$\omega_{1}$	$T_{1,\text{DNP}}$ (s)	Enhancement*
$R6_{4}^{\pm 1}$	3	90_x_270_−x_	1.5$\omega_{0I}$	9.7	333
Riffled $R6_{4}^{\pm 1}$	3	90_y_180_x_90_y_	1.5$\omega_{0I}$	9.7	350
$C7_{13}^{\pm 1}$	2	90_x_360_−x_270_x_	1.077$\omega_{0I}$	10.1	293
NOVEL	1	–	–	∼8†	330

* This is the maximum enhancement factor at equilibrium, i.e., the DNP spectrum was recorded with a DNP buildup time > $3T_{1,\text{DNP}}$, *h* = 32,768, and then extrapolated to infinite time.

† Taken from the literature reported by Mathies *et al.* (*37*) on similar experimental conditions.

**Fig. 3.**
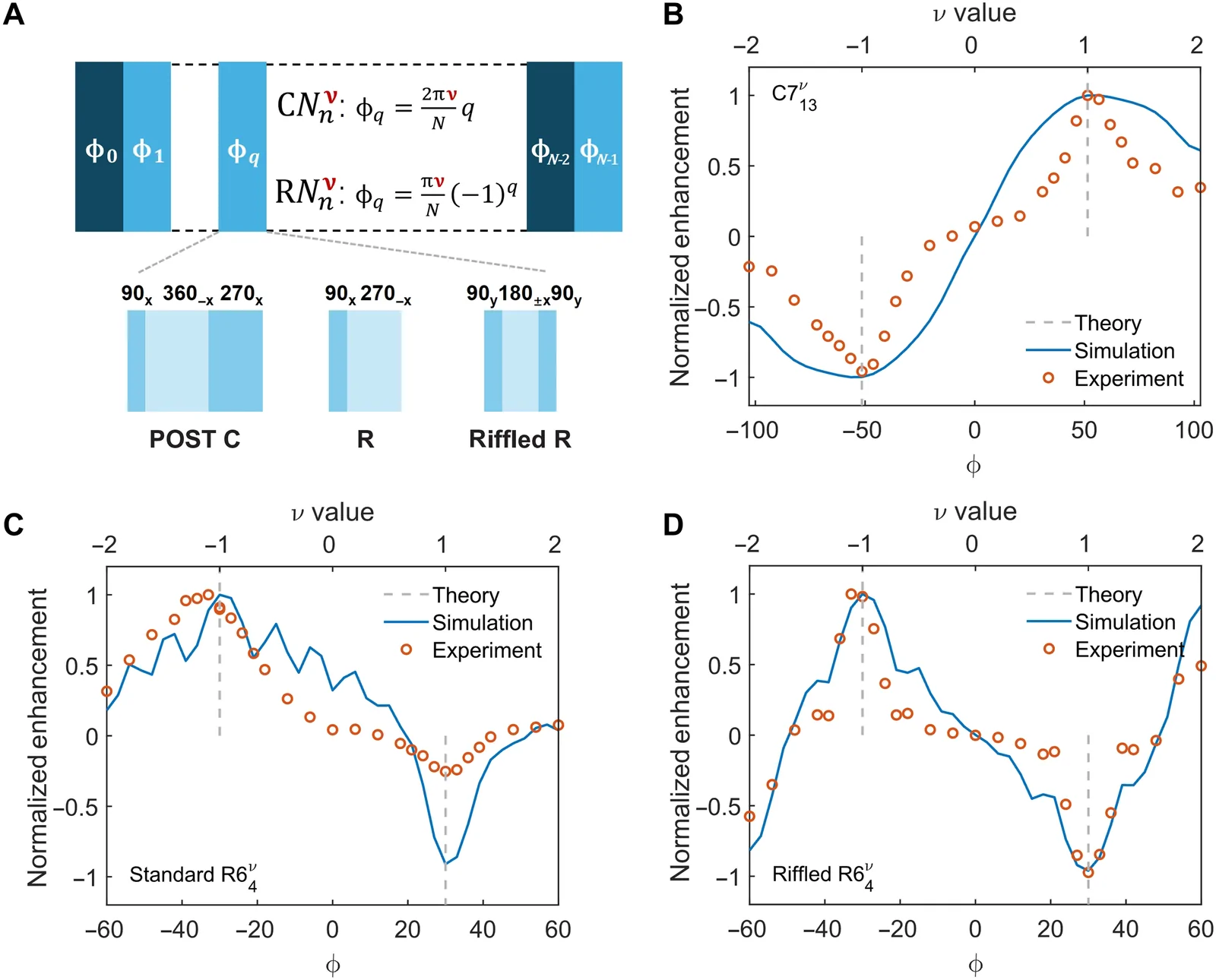
Phase (ν) profile of symmetry-based pulsed DNP. (**A**) Schematic diagram of symmetry-based pulses and three different basic elements. (**B** to **D**) Numerically simulated (blue solid line) and experimentally observed (red circle) normalized ν profiles of (B) $C7_{13}^{\nu }$, (C) $R6_{4}^{\nu }$, and (D) riffled $R6_{4}^{\nu }$.

In principle, each pulse element with an incrementing phase $\phi_{q}$ (Fig. 1) could be a simple 2π pulse for $CN_{n}^{\nu }$ sequences; however, these pulses typically exhibit poor tolerance to resonance offsets and RF inhomogeneity. We instead used the POST composite 2π pulse ($90_{\phi }360_{\phi +\pi }270_{\phi }$; Fig. 3A) with a duration of two 2π pulses, which has been shown to provide superior robustness against experimental artifacts while maintaining high scaling factors. For the $C7_{13}^{1}$ sequence, the *N* = 7 POST elements are synchronized within *n* = 13 ^1^H Larmor periods [i.e., 13/(14.86MHz)=875ns], necessitating a Rabi frequency of $\omega_{1}=\left(2N\omega_{0I}\right)/n=16\;$ MHz. Given the maximum Rabi frequency of 30 MHz achievable with our X-band traveling-wave tube (TWT) microwave amplifier, we selected several representative sequences, summarized in Table 2. An efficient composite pulse ($90_{\phi }270_{\phi +\pi }$; Fig. 3A) was used as the basic *R* element. Consistent with the matching conditions in Table 1, $C7_{13}^{1}$ or $R6_{4}^{-1}$ yielded positive DNP enhancements, whereas $C7_{13}^{-1}$ or $R6_{4}^{1}$ produced negative DNP enhancements. To visualize these selection rules for DNP, we adapt the “space-spin selection diagram” into the DNP matching diagram (Fig. 1B). In DNP, the matching condition corresponds to the synchronization of the modulation frequencies of two rank 1 spin tensors related to electron and nuclei. Consequently, the allowed values of *m* and μ are −1, 0, and 1. For positive DNP, both *m* and μ should be nonzero and have the same sign. On the basis of the DNP matching diagram, one can therefore identify which combinations of ν and *n* satisfy the DNP matching condition.

Consistent with our theoretical predictions, we recorded a positive DNP enhancement of ε ∼ 333 (Fig. 2B) using $R6_{4}^{-1}$ with optimized parameters (*l, h,* and $\omega_{1}$). As a proof of principle, we also implemented a “riffled” version of $R6_{4}^{-1}$ (*12*) and the $C7_{13}^{1}$ sequence (Fig. 3A). These sequences yielded comparable DNP enhancements of ε ∼ 293 to 350, which are similar to, or slightly exceed, that of NOVEL (ε ∼ 330)—often known as the gold standard for pulsed DNP sequence (Fig. 2C).

To confirm that the hyperpolarization is explicitly mediated by the symmetry-based sequences rather than by competing DNP mechanisms, we recorded DNP “phase profiles” (Fig. 3, B to D). In these experiments, the DNP enhancement factor was measured as a function of the phase $\phi_{1}=\frac{2\text{πν}}{N}$ ($CN_{n}^{\nu }$) or $\frac{\text{πν}}{N}$ ($RN_{n}^{\nu }$). Consistent with our theoretical framework, we observed DNP maxima at $\phi_{1}$ ≈ ±50° (Fig. 3B), which shows excellent agreement with the theoretical prediction of $\phi_{1}=\left(\pm 1\;\times \;360{}^{\circ}\right)/7∼\pm \;51.4{}^{\circ}$ (or ν=±1) for $C7_{13}^{\pm 1}$. Similarly, experimental results using (riffled) $R6_{4}^{\pm 1}$ exhibited maximum enhancements at $\phi_{1}\approx \pm \;30{}^{\circ}=\pm \;180{}^{\circ}/6$ (ν=±1), again matching our theoretical predictions (Fig. 3C). Furthermore, these experimental phase profiles largely agree with numerical simulations, with differences on enhancement likely arising from the isolated three-spin model used for simulation (see Materials and Methods), which ignores the fast relaxation of nearby nuclei and spin diffusion processes (fig. S2). The robust agreement between theory, experiments, and simulations underscores the power of identifying mathematical symmetries across magnetic resonance disciplines. It demonstrates that advanced NMR MAS methodologies can be adapted to the static DNP and EPR regimes with minimal modification, providing a streamlined route for pulse sequence development in magnetic resonance at various fields, provided that the necessary instrumentation is available.

**Fig. 4.**
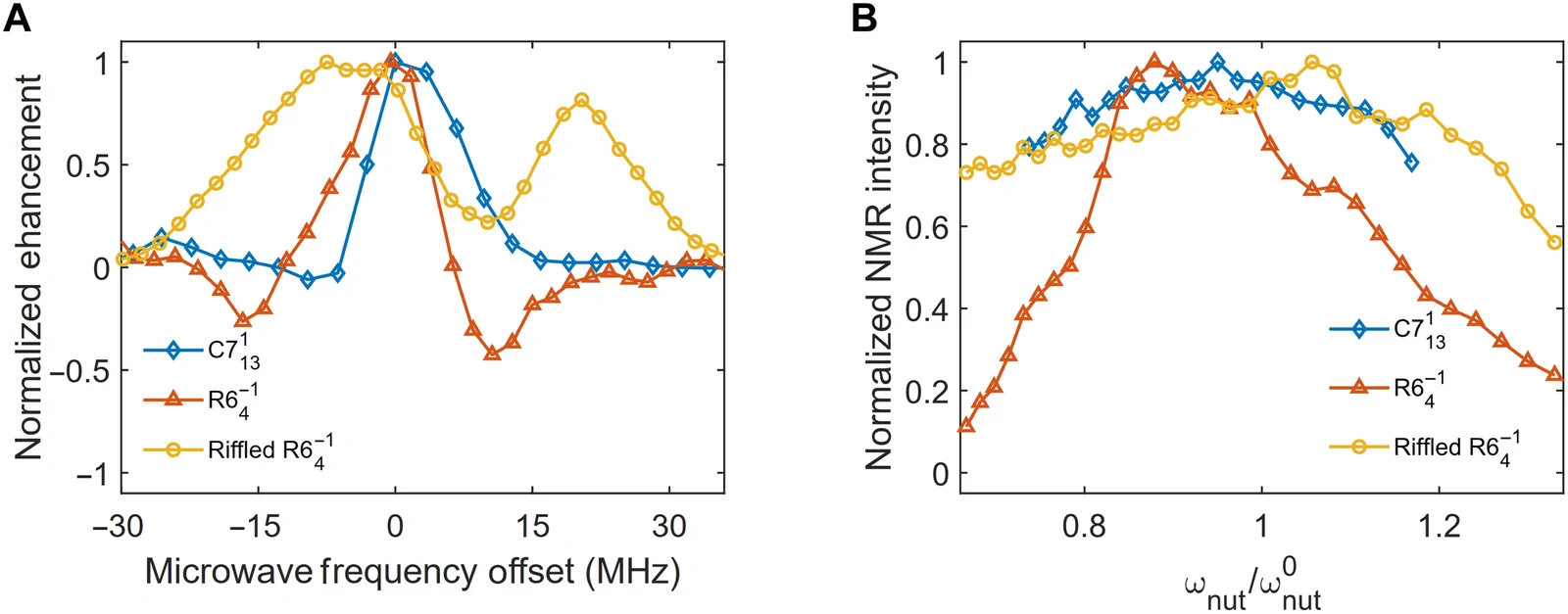
Robustness of symmetry-based pulsed DNP. (**A**) DNP frequency profiles of $C7_{13}^{1}$, $R6_{4}^{-1}$, and riffled $R6_{4}^{-1}$. The microwave frequency offset is calculated with reference to the EPR frequency of OX071 at 0.35 T. (**B**) DNP nutation frequency profiles. The theoretical matching Rabi frequency of the microwave is defined as $\omega_{\text{nut}}^{0}$, and $\omega_{\text{nut}}$ is the microwave Rabi frequency used in the experiment.

We further evaluated the robustness of the three symmetry-based sequences against resonance offsets and *B*_1_ field inhomogeneity. The riffled $R6_{4}^{-1}$ sequence demonstrated the highest tolerance to both frequency offsets (Fig. 4A) and *B*_1_ field deviations (Fig. 4B) compared to the other investigated sequences, as predicted by the simulation (fig. S3). This superior performance is consistent with its previously reported excellence in a vastly different application: singlet-triplet conversion (*12*). This observation underscores the power of a unified symmetry-based framework; it allows a sequence established in one specialized subfield (e.g., solution-state NMR singlet-triplet conversion) to be directly translated to another (e.g., pulsed DNP) with minimal modification while retaining its inherent robustness. Although a formal theoretical derivation for this specific adaptation is beyond the scope of this work, a rigorous analysis of the underlying mechanism will be presented in a forthcoming study.

### Symmetry-based pulsed EPR sequence

Since DNP is fundamentally an electron-nuclear process, we hypothesized that the electron-detected counterparts of these symmetry-based sequences could provide complementary information inaccessible via standard DNP-NMR. For instance, monitoring the depletion of the electron polarization during the DNP block could provide deeper mechanistic insights into polarization transfer. Moreover, these electron-detected experiments could be adapted to probe hyperfine interactions, serving a role similar to ENDOR or ESEEM spectroscopies (*23*). Conventionally, Davies-ENDOR is used for detecting large hyperfine couplings, whereas smaller (pseudo-secular) couplings are typically measured using Mims-ENDOR or one-dimensional (1D)– and 2D-ESEEM techniques (*24*). However, under specific experimental conditions, determined by the magnetic field strength and relaxation rates, there exists an intermediate regime of hyperfine interactions where neither ENDOR nor ESEEM operates optimally. In this context, symmetry-based sequences could offer a complementary approach by allowing the scaling factors $[\kappa_{1m1\mu }\left(N,n,\nu \right)$; Eq. 10] to be tuned for specific coupling strengths. This flexibility enriches the pulsed EPR toolkit for distance measurements.

**Fig. 5.**
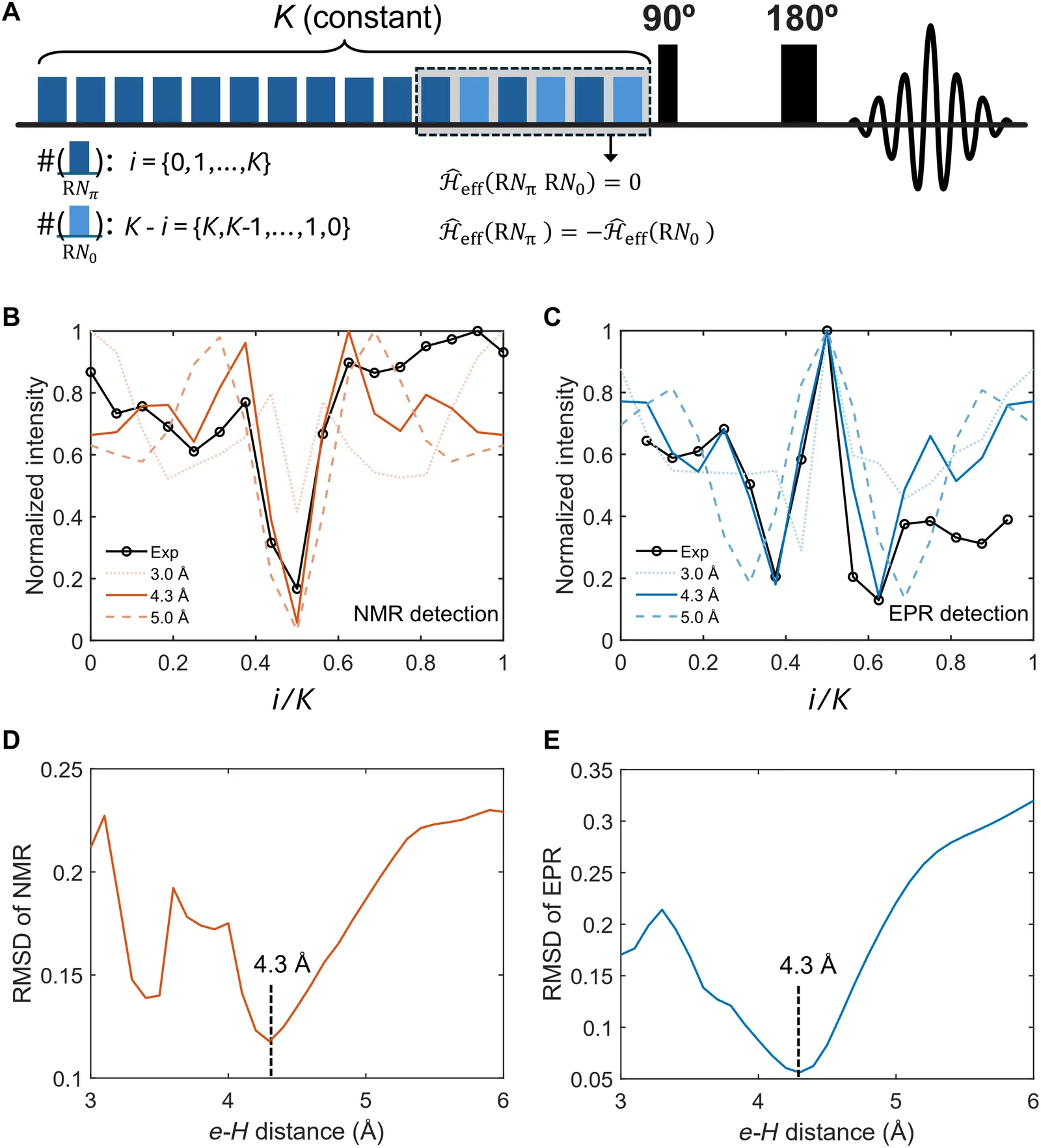
Constant-time experiments based on symmetry-based pulses. (**A**) Schematic diagram of the pulse sequence. The riffled $R6_{4}^{-1}$ sequence is denoted as R*N*_0_, while R*N*_π_ represents the same riffled $R6_{4}^{-1}$ block with a global π phase shift applied to all pulses. Either R*N*_0_ or R*N*_π_ can drive DNP, but because the effective Hamiltonians generated by R*N*_0_ and R*N*_π_ have opposite signs, a pair of these two blocks produces a net zero effective Hamiltonian. The number of R*N*_π_ and R*N*_0_ blocks is given by *i* and (*K − i*), respectively. The sum of the blocks is denoted *K*, which corresponds to the total mixing time and is kept constant. The polarization transfer process is controlled by the proportion of R*N*_π_ blocks (*i/K*) and monitored using either (**B**) NMR or (**C**) EPR detection. A more intuitive illustration of the pulse sequence is shown in fig. S4. The polarization transfer curves were fitted using a three-proton-one-electron model with the same electron-nuclear distances. The root mean square deviation (RMSD) between simulation and experiment was calculated as a function of the electron-nuclear distance with (**D**) NMR and (**E**) EPR detection.

To probe the hyperfine interaction, we designed a constant-time experiment based on symmetry-based pulses (Fig. 5A) that monitors the transfer of polarization between electron and nuclear spins. The experiment was implemented in a constant-time manner so that relaxation contributes equally at all time points, enabling more accurate determination of the hyperfine interaction. This approach is analogous to constant-time DIPSHIFT experiments in MAS NMR (*25*).

The sequence is constructed from a symmetry-based pulse block, riffled $R6_{4}^{1}$, denoted as [R*N*_0_] ($\left\{90_{60}180_{330}90_{60}90_{300}180_{210}90_{300}\right\}\times 3$). Applying a global π phase shift to all pulses in [R*N*_0_] produces the corresponding block [R*N*_π_] ($\left\{90_{240}180_{150}90_{240}90_{120}180_{30}90_{120}\right\}\times 3$), which generates an effective Hamiltonian with the opposite sign relative to [R*N*_0_] (*26*). As a result, a pair of adjacent [R*N*_0_] and [R*N*_π_] blocks produces zero net polarization transfer. The total number of blocks of [R*N*_0_] and [R*N*_π_], *K*, determines the overall mixing time, which is kept constant, while the polarization transfer dynamics are controlled by the proportion of [R*N*_π_] blocks *i/K*. The proportion increases by converting selected [R*N*_0_] blocks to [R*N*_π_] in an interleaved manner (fig. S4). This interleaving strategy minimizes the influence of relaxation effects and extends the dynamic range of evolution.

Using an integrated EPR/NMR setup (fig. S5), the dynamics of polarization transfer were monitored by the dual-channel acquisition (Fig. 5, B and C). As *i/K* increases from 0 to 0.5, the effective polarization transfer time decreases due to progressive cancellation between [R*N*_0_] and [R*N*_π_] blocks. At *i/K* = 0.5, the effective Hamiltonians cancel completely, resulting in zero net polarization transfer between electron and nuclei, which corresponds to the minimum DNP NMR signal and maximum EPR signal. As *i/K* increases beyond 0.5, [R*N*_π_] blocks dominate the polarization transfer, and the effective polarization transfer time increases again.

This anticorrelated relationship between EPR and NMR intensities provides complementary insights into the electron-nuclear transfer mechanism. Theoretically, the “backward” (^1^H → e^−^: *i/K* from 0 to 0.5) and the “forward” (e → ^1^H: *i/K* from 0.5 to 1.0) transfer curves should be perfectly symmetric. However, we observed a slight experimental asymmetry, likely due to imperfect phase control (IQ imbalance) in the microwave AWG source and decoherence from relaxation (fig. S6). Despite this imperfection, the positions of the extrema remained symmetrically distributed. To evaluate the role of the nuclear spin diffusion between the radical-proximal protons and the bulk nuclei (*27*–*29*), we repeated the EPR experiment while applying CW saturation RF pulses to the bulk ^1^H NMR signal. While this saturation decreased very slightly the EPR signal intensity, the overall profile remained unchanged (fig. S7). These observations imply that while minor spin diffusion occurs between the radical-proximal protons and the bulk ^1^H spins, it is not sufficiently pronounced, given the microsecond-scale mixing times, to alter the profile of the transfer curve. Conversely, active decoupling of the bulk ^1^H reservoir enhances the dynamic range of the transfer curve. This enhancement is critical as it facilitates a more accurate determination of the hyperfine interactions within the target spin system, effectively isolating the local electron-nuclear dynamics from the bulk environment.

Subsequently, we performed numerical simulations (see the section “Simulation of DNP and EPR”) using a simplified three-nucleus-one-electron model to determine the electron-nuclear distance. This approach was adopted because incorporating all 24 trityl protons in full numerical simulations is not computationally feasible (*30*). The simulated dephasing-refocusing curves (Fig. 5, B and C) using a distance of ∼4.3 Å provided the best fit to the experimental data, yielding the lowest root mean square deviation (RMSD; Fig. 5, D and E). This result is not very far from the closest electron-nuclear distance (5.1 Å) predicted by density functional theory simulations (*27*). While the RMSD analysis for the NMR-detected data exhibited a second local minimum at 3.4 Å, the simulated curve for this shorter distance failed to capture the characteristic oscillations observed experimentally (fig. S8) and did not correspond to electron-detected data. We emphasize that the current results on trityl provide merely a rough estimation of electron-nuclear coupling strengths. A more rigorous characterization using an isolated spin system is required to better characterize the performance of the sequences, but such a study lies beyond the scope of this work.

To further demonstrate the versatility of our theoretical framework, we repeated the distance measurements and simulations with the $R4_{3}^{1}$ sequence. These cross-verification experiments yielded an identical electron-nuclear distance of 4.3 Å (fig. S9), underscoring the reliability and quantitative potential of symmetry-based pulse sequences for the characterization of electron-nuclear interactions. Although the relatively sparse time-domain sampling could be improved by choosing a sequence with a smaller scaling factor (e.g., $R6_{11}^{-2}$; fig. S10), the maximum TWT gate time of the microwave amplifier restricts the number of symmetry blocks that can be applied. In principle, this limitation could be improved at higher magnetic fields, where the shorter nuclear Larmor period decreases the pulse-block duration and allows denser sampling within the gate time constraint.

Besides that, it is known that the presence of electron-electron couplings or radical concentration could affect the electron-nuclear distance measurement. Inspired by the fact that the symmetry-based sequences were demonstrated in MAS NMR for heteronuclear dipolar recoupling with simultaneous decoupling of homonuclear interaction (*31*), we hypothesized that the symmetry-based constant-time experiment demonstrated earlier (Fig. 5) may suppress electron-electron couplings. As a proof of principle, numerical simulations were performed on a three-spin e-e-^1^H system (fig. S11), and the results show that the symmetry-based EPR sequence remains robust when the electron-electron dipolar coupling is as high as 6.5 MHz, which corresponds to a concentration of ∼200 mM. While the suppression of electron-electron couplings can be analytically described using higher-order average Hamiltonian theory, such a derivation lies beyond the scope of this study (*18*).

We have demonstrated that the theoretical framework of symmetry-based pulse sequences, originally developed for MAS NMR, can be successfully translated to the static pulsed DNP and EPR regimes by synchronizing the microwave irradiation duration with the nuclear Larmor period rather than the mechanical rotor period. Our results on trityl OX071 at 0.35 T confirm that sequences such as $C7_{13}^{1}$ and (riffled) $R6_{4}^{-1}$ achieve DNP enhancements comparable to the “gold standard” NOVEL sequence while offering greater robustness against microwave power inhomogeneities and resonance offsets. The rigorous agreement between experimental phase profiles and numerical simulations confirms that the polarization transfer is precisely mediated by the symmetry-based Hamiltonian.

Beyond hyperpolarization, we extended this methodology to pulsed EPR to estimate effective hyperfine interactions and probe local electron-nuclear environments. This approach offers a considerable flexibility in “tuning” the scaling factors for specific experimental needs via symmetry integers (*N*, *n*, and ν), basic element selection, and various supercycling strategies, and all of which have been extensively developed in the MAS NMR literature. While we have experimentally validated the pulsed DNP technique on a model system (trityl), we anticipate that this methodology can be extended to NV centers and photoexcited triplet systems (e.g., pentacene and its derivatives) using comparable low-field instrumentation (*32*, *33*).

This framework provides distinct methodological advantages; unlike ENDOR, these symmetry-based, electron-detected experiments do not require simultaneous RF irradiation. Furthermore, the first-order Hamiltonian of the symmetry-based methods is theoretically field independent. This implies that their performance should remain consistent at various fields, if the required hardware is readily available. This constancy contrasts favorably with ESEEM, whose sensitivity is governed by forbidden transition probabilities that decrease at higher external *B*_0_ fields.

Ultimately, the introduction of symmetry-based designs into DNP and EPR provides a versatile extension to the magnetic resonance toolkit, offering additional options for structural analysis or quantum sensing in complex, disordered, or dilute paramagnetic systems. We would like to emphasize that further comparative studies are necessary to fully evaluate the relative performance of these sequences against standard DNP and EPR sequences (ENDOR, ESEEM, etc.). However, the current manuscript is intended as a foundational proof of principle, establishing the adaptability of MAS NMR symmetry rules to pulsed static DNP and EPR.

## MATERIALS AND METHODS

### DNP and EPR experiments at 0.35 T

The experiments were performed on 5 mM trityl OX071 (or *d*_24_-OX063) in a mixture of d_8_-glycerol/D_2_O/H_2_O (6:3:1 by volume). The OX071 trityl radical was synthesized according to published protocols (fig. S12) (*34*). Measurements were performed at 80 K using a 0.35 T Bruker Elexsys E580 X-band spectrometer. The system was equipped with a 1-kW TWT amplifier and a 1.6 GS/s arbitrary waveform generator. NMR acquisition was performed using a standard Bruker AVANCE II console with a 500-W RF amplifier connected to the ENDOR probe via a custom-build tuning and matching network. The presaturation on ^1^H (Fig. 2A) was achieved using 512° × 30° pulses spaced by a delay of 200 μs. The ^1^H signal was acquired using spin echo with τ = 20 μs with an RF Rabi frequency of 113.6 kHz. All the experiments were run under 3479.780 G, with an on-resonance microwave frequency of 9.7564 GHz and ^1^H Larmor frequency of 14.86 MHz. The EPR signal was acquired by a spin echo with τ = 600 ns and an electron nutation frequency of 15.6 MHz. To ensure synchronization, all microwave pulse durations were set as integer multiples of the AWG time resolution (625 ps), and the magnetic field *B*_0_ was precisely adjusted to synchronize the symmetry-based pulse durations with integer multiples of the nuclear Larmor period.

### Simulation of DNP and EPR

Numerical simulations were performed using the Spinach library for MATLAB (*35*). Symmetry-based DNP was modeled using a three-spin system consisting of two electrons and one proton. The isotropic hyperfine interaction was omitted, and the electron-nuclear distance was set to 4.3 Å. The parameters and simplified spin system model used in our simulations were adapted directly from our previously published work (*27*, *30*). The hyperfine interaction Euler angles were defined as (0°, 0°, 0°) and (70°, 90°, 0°) for the principal axis systems (PAS). For the electron, a g-tensor of [2.0046, 2.0038, 2.0030] was used with Euler angles of (0°, 40°, 0°). Relaxation parameters were set as *T*_1e_ = 1 ms, *T*_2e_ = 2 μs and *T*_1n_ = 13 s, *T*_2n_ = 1 ms for the electron and ^1^H nuclei, respectively. The symmetry-based DNP phase (ν) profile was simulated in the same three-spin system with either 3 or 2 buildup blocks for the $RN_{n}^{\nu }$ or $CN_{n}^{\nu }\;$ sequence, respectively, as used experimentally. The enhancement was taken when the simulated DNP buildup curves reach the plateau. The constant-time symmetry-based EPR experiments were fitted using a model comprising one electron and three protons. This model used the same electron parameters but incorporated three hyperfine interactions with Euler angles of (0°, 60°, 0°), (120°, 60°, 0°), and (240°, 60°, 0°) for their PAS. All simulation scripts are hosted and publicly accessible via Zenodo (https://zenodo.org/records/19122751).
