# Supplementary material for: Symmetry-based pulsed DNP and EPR sequences
Source: Sci Adv. 2026 Sep 16;12(38):eaef4949. doi: 10.1126/sciadv.aef4949 (PMC13580538; doi:10.1126/sciadv.aef4949)
Supplement: Supplementary file 1 [file sciadv.aef4949_sm.pdf]

Science Advances
AAAS

Supplementary Materials for

Symmetry-based pulsed DNP and EPR sequences

Zhenfeng Pang *et al.*

Corresponding author: Kong Ooi Tan, kong-ooi.tan@ens.psl.eu

Sci. Adv. **12**, eaef4949 (2026)
DOI: 10.1126/sciadv.aef4949

This PDF file includes:

Supplementary Text S1
Figs. S1 to S12

Supplementary Text

Text S1. Rewrite the Hamiltonian using irreducible spherical tensors

Hyperfine coupling consists of the isotropic part and the dipolar part. We can write the Cartesian tensor of hyperfine coupling $\boldsymbol{A}$ as:

$$\boldsymbol{A} = A^{\text{iso}}\mathbf{1} + \boldsymbol{T} = \begin{pmatrix} A_{\text{xx}} & A_{\text{xy}} & A_{\text{xz}} \\ A_{\text{yx}} & A_{\text{yy}} & A_{\text{yz}} \\ A_{\text{zx}} & A_{\text{zy}} & A_{\text{zz}} \end{pmatrix} \tag{S1}$$

where A^{iso} is the isotropic hyperfine coupling constant, **1** is the identity matrix, and *T* is a traceless anisotropic tensor describing the dipolar part. Because the dipolar coupling is traceless and symmetric, the elements of hyperfine coupling tensor satisfy the equations:

$$\begin{gathered} A_{\text{xx}} + A_{\text{yy}} + A_{\text{zz}} = 3A^{\text{iso}} \\ A_{\text{xy}} = A_{\text{yx}} \\ A_{\text{xz}} = A_{\text{zx}} \\ A_{\text{yz}} = A_{\text{zy}} \end{gathered} \tag{S2}$$

Then we can calculate the elements of the second-rank spherical spatial tensor:

$$\begin{gathered} A_{00} = -\frac{1}{\sqrt{3}}\left(A_{\text{xx}} + A_{\text{yy}} + A_{\text{zz}}\right) = -\sqrt{3}A^{\text{iso}} \\ A_{10} = -\frac{i}{\sqrt{2}}\left(A_{\text{xy}} - A_{\text{yx}}\right) = 0 \\ A_{1,\pm 1} = -\frac{1}{2}\Big(A_{\text{zx}} - A_{\text{xz}} \pm i\left(A_{\text{zy}} - A_{\text{yz}}\right)\Big) = 0 \\ A_{20} = \frac{1}{\sqrt{6}}\Big(3A_{\text{zz}} - \left(A_{\text{xx}} + A_{\text{yy}} + A_{\text{zz}}\right)\Big) = \frac{3}{\sqrt{6}}\left(A_{\text{zz}} - A^{\text{iso}}\right) \\ A_{2,\pm 1} = \mp\frac{1}{2}\Big(A_{\text{xz}} + A_{\text{zx}} \pm i\left(A_{\text{yz}} + A_{\text{zy}}\right)\Big) = \mp\left(A_{\text{zx}} \pm iA_{\text{zy}}\right) \\ A_{2,\pm 2} = \frac{1}{2}\left(A_{\text{xx}} - A_{yy} \pm 2iA_{\text{xy}}\right) \end{gathered} \tag{S3}$$

In order to consider the influence of microwaves on the electron, we decompose the second-rank spherical spin-tensor operators in two first-rank operators as:

$$\begin{gathered} \hat{T}_{00} = \frac{1}{\sqrt{3}}\left(\hat{T}_{1,-1}^{(I)}\hat{T}_{1,1}^{(S)} + \hat{T}_{1,1}^{(I)}\hat{T}_{1,-1}^{(S)} - \hat{T}_{10}^{(I)}\hat{T}_{10}^{(S)}\right) \\ \hat{T}_{20} = \frac{1}{\sqrt{6}}\left(2\hat{T}_{10}^{(I)}\hat{T}_{10}^{(S)} + \hat{T}_{1,-1}^{(I)}\hat{T}_{1,1}^{(S)} + \hat{T}_{1,1}^{(I)}\hat{T}_{1,-1}^{(S)}\right) \\ \hat{T}_{2,\pm 1} = \frac{1}{\sqrt{2}}\left(\hat{T}_{1,\pm 1}^{(I)}\hat{T}_{1,0}^{(S)} + \hat{T}_{1,0}^{(I)}\hat{T}_{1,\pm 1}^{(S)}\right) \\ \hat{T}_{2,\pm 2} = \hat{T}_{1,\pm}^{(I)}\hat{T}_{1,\pm 1}^{(S)} \end{gathered} \tag{S4}$$

where spin *S* is electron and spin *I* is nucleus. When the secular approximation is satisfied, i.e., the

electron Larmor frequency is much larger than hyperfine coupling, we can drop the terms without $\hat{T}_{1,0}^{(S)}$ in the electron rotating frame:

$$\hat{T}_{00} \rightarrow -\frac{1}{\sqrt{3}}\hat{T}_{1,0}^{(I)}\hat{T}_{1,0}^{(S)}$$
$$\hat{T}_{20} \rightarrow \frac{2}{\sqrt{6}}\hat{T}_{1,0}^{(I)}\hat{T}_{1,0}^{(S)}$$
$$\hat{T}_{2,\pm1} \rightarrow \frac{1}{\sqrt{2}}\hat{T}_{1,\pm1}^{(I)}\hat{T}_{1,0}^{(S)} \tag{S5}$$
$$\hat{T}_{2,\pm2} \rightarrow \text{neglect}$$

and we can write down the Hamiltonian of hyperfine coupling using irreducible spherical tensor:

$$\hat{\mathcal{H}}_{\text{hf}} = A_{00}\hat{T}_{00} + \sum_{m=-2}^{2}(-1)^m A_{2,m}\hat{T}_{2,-m}$$

with secular approximation:

$$\hat{\mathcal{H}}_{\text{hf}} = \left(-\frac{1}{\sqrt{3}}A_{00} + \frac{2}{\sqrt{6}}A_{20}\right)\hat{T}_{1,0}^{(I)}\hat{T}_{1,0}^{(S)} - \frac{1}{\sqrt{2}}A_{2,\pm1}\hat{T}_{1,\mp1}^{(I)}\hat{T}_{1,0}^{(S)} \tag{S6}$$
$$= \sum_{m=-1}^{1}(-1)^m\omega_m\hat{T}_{1,-m}^{(I)}\hat{T}_{1,0}^{(S)}$$

with the coefficients:

$$\omega_0 = -\frac{1}{\sqrt{3}}A_{00} + \frac{2}{\sqrt{6}}A_{20} = A_{zz}$$
$$\omega_{\pm1} = \frac{1}{\sqrt{2}}A_{2,\pm1} = \mp\frac{1}{\sqrt{2}}(A_{\text{zx}} \pm iA_{\text{zy}}) \tag{S7}$$

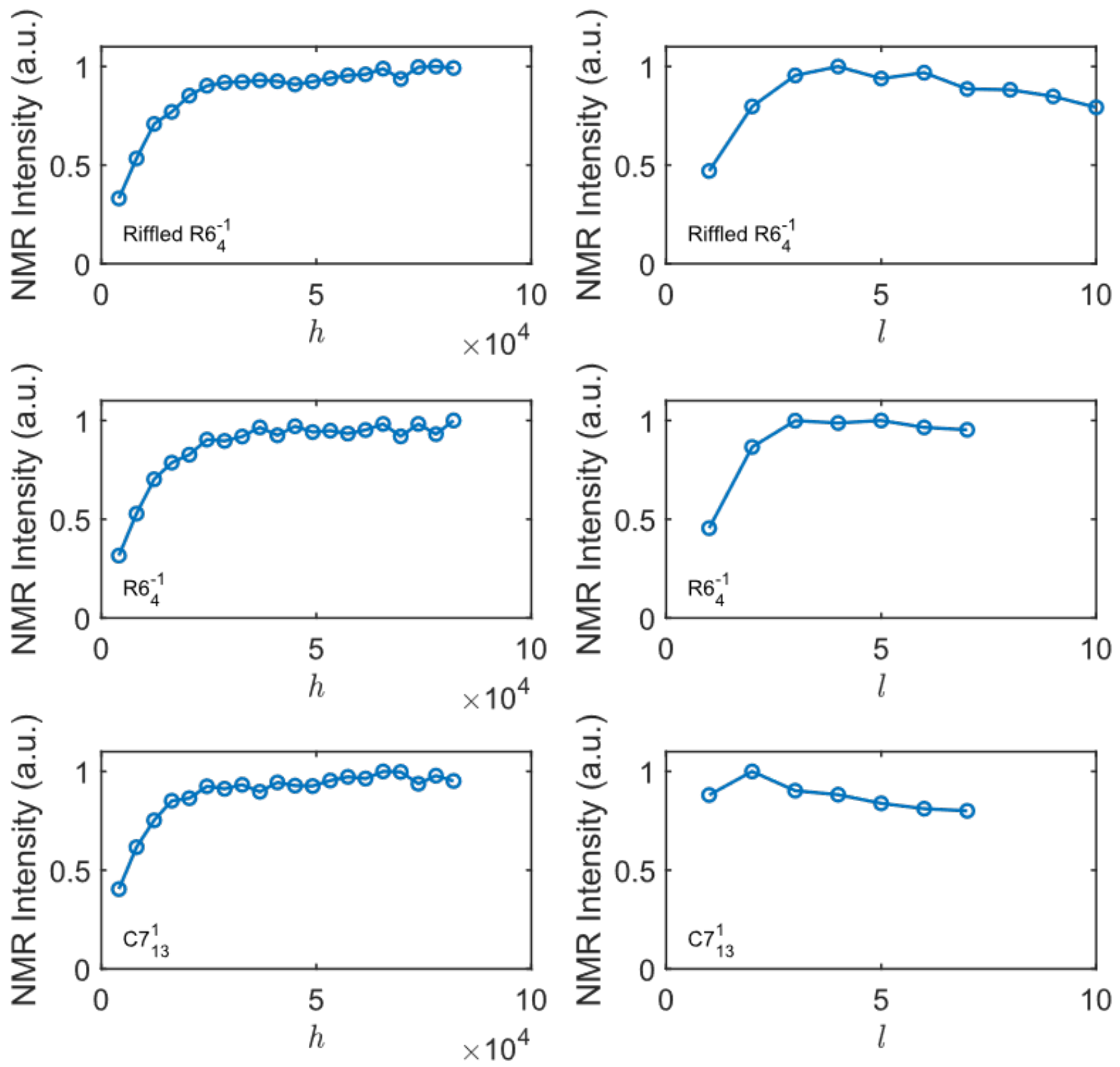

Fig. S1 DNP build up versus *h* and *l* values using different symmetry-based pulses

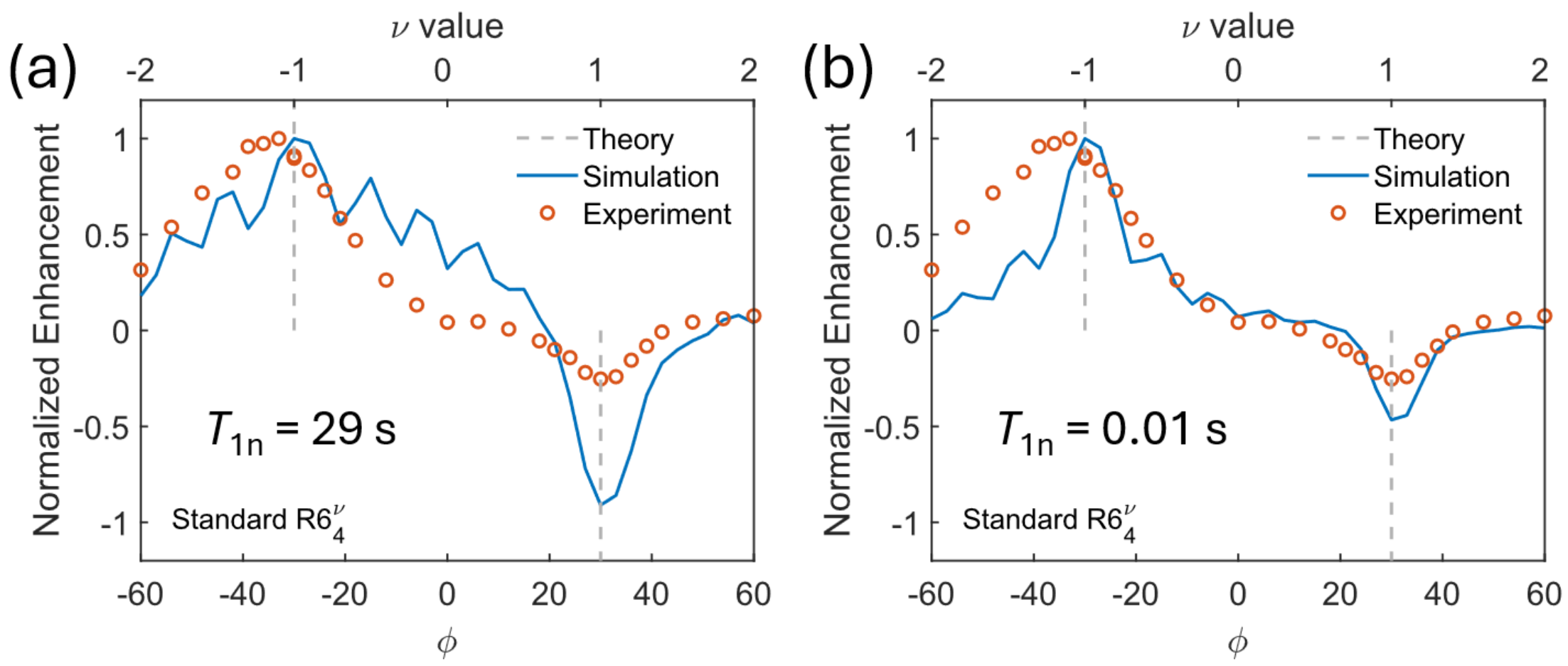

Fig. S2 Phase profiles with different nuclear T_1 relaxation time. (a) 29 s. (b) 0.01s. The simulated matching condition becomes narrower when a shorter nuclear T_1 is used. In a real sample, polarization is first transferred from the electron to nearby nuclei and subsequently transferred to the bulk nuclei via spin diffusion. When the ν value deviates from the matching condition, the polarization transfer rate from the electron to the nearby nuclei decreases. As a result, a larger fraction of the polarization relaxes on the nearby nuclei before being transported to the bulk. In the simulations, however, the nuclear relaxation rate is set to the value measured for bulk protons, whose relaxation is much slower than the polarization transfer rate. This allows polarization to accumulate efficiently. In contrast, in the real sample, the DNP enhancement is further limited by the relatively fast relaxation of the nuclei in close proximity to the electron.

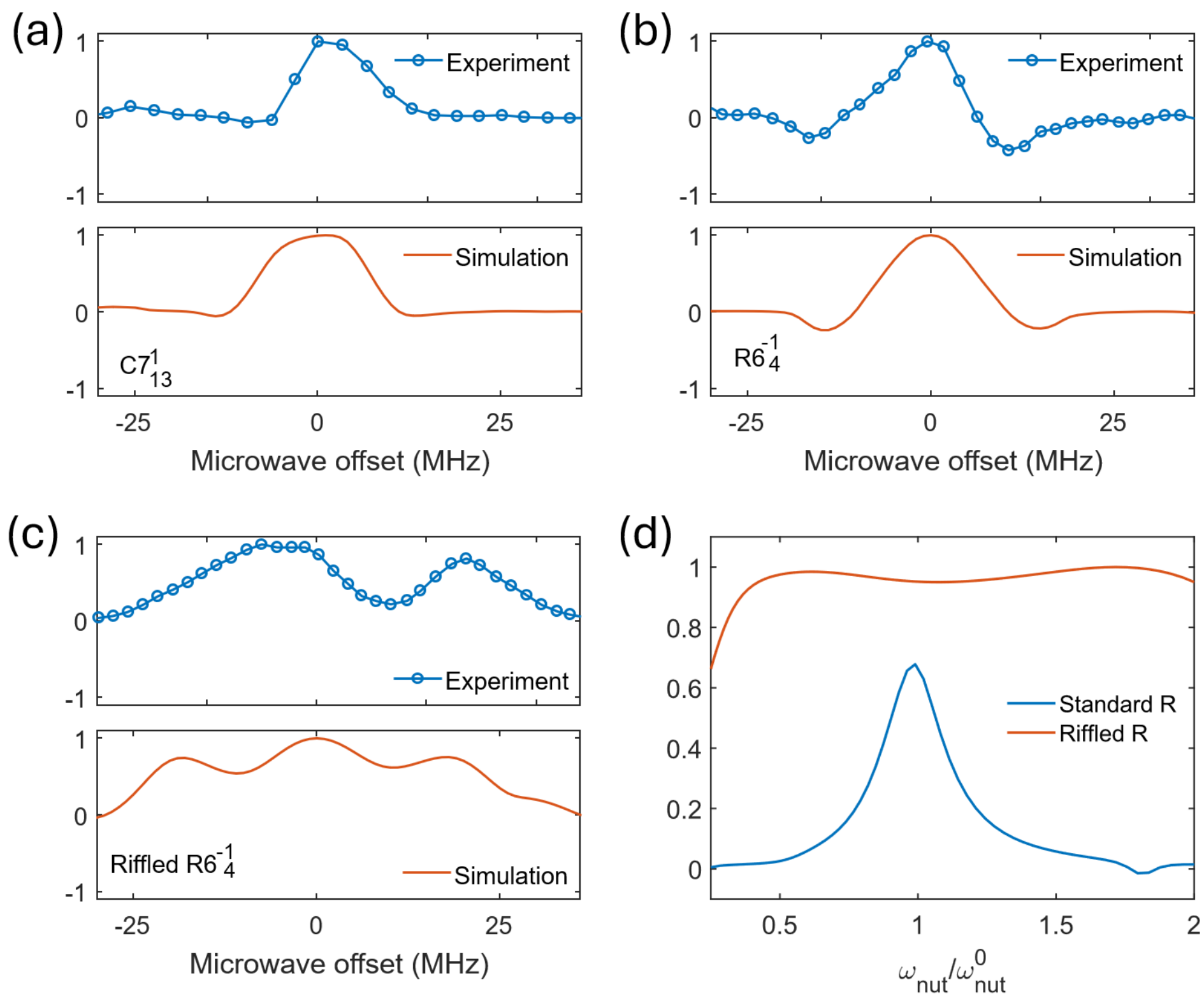

Fig. S3 Simulation for the robustness of symmetry-based DNP. (a-c) microwave offset for $C7_{13}^{1}$, standard $R6_4^{-1}$, and riffled $R6_4^{-1}$. (d) Simulated tolerance on B_1 field inhomogeneity for standard $R6_4^{-1}$ and riffled $R6_4^{-1}$.

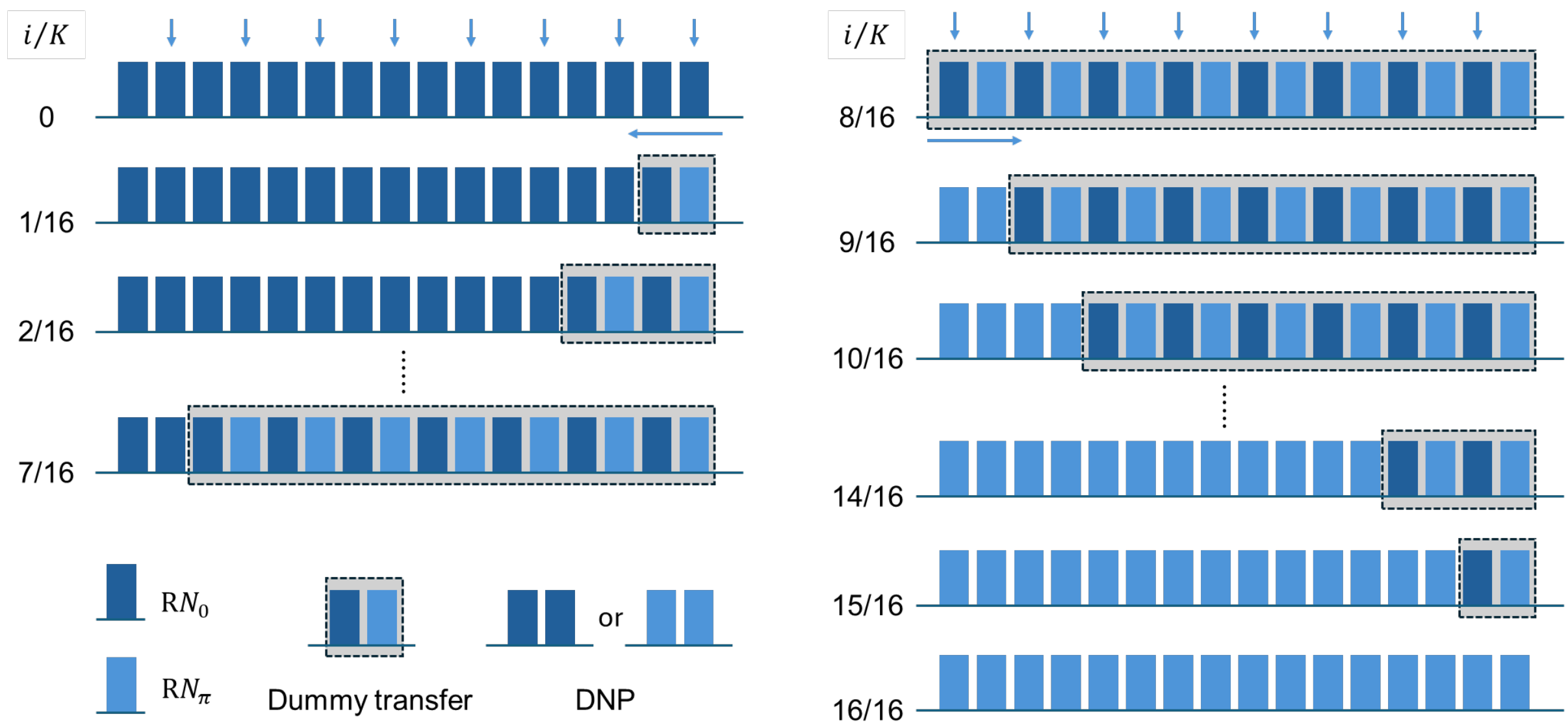

Fig. S4 Details of constant-time pulses. For riffled $R6_4^{-1}$, the basic element is $90_{90}180_{0}90_{90}$. RN_0 is defined with $\{90_{60}180_{330}90_{60}90_{300}180_{210}90_{300}\} \times 3$, and RN_π with $\{90_{240}180_{150}90_{240}90_{120}180_{30}90_{120}\} \times 3$. Note that K = 16 blocks have been chosen here, and K can be changed to alter the total mixing time. The minimum time increment is fixed with the duration span by two $[RN_{0/\pi}]$ blocks.

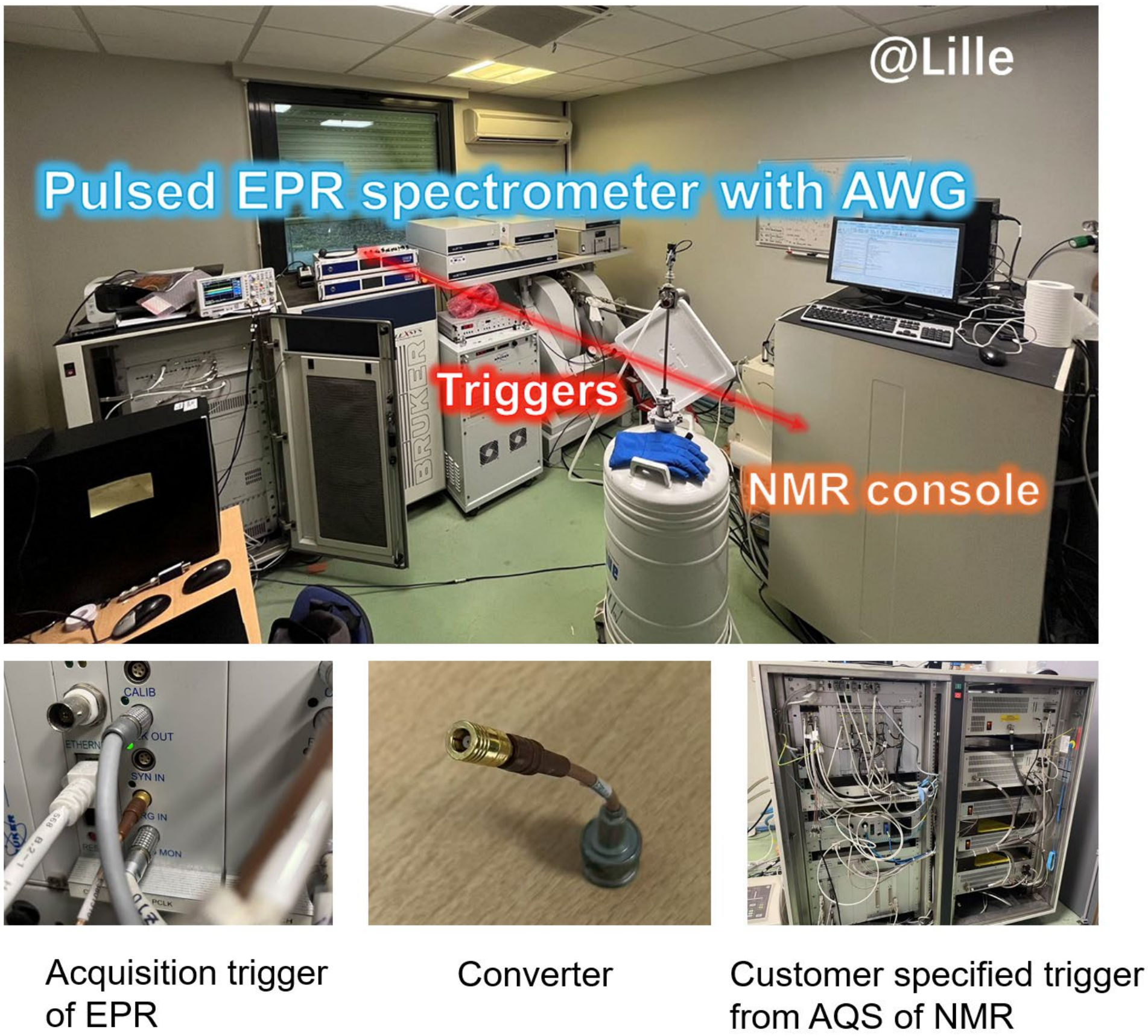

Fig. S5 Pulsed DNP setup. The EPR console is triggered by the NMR console through the TTL signal.

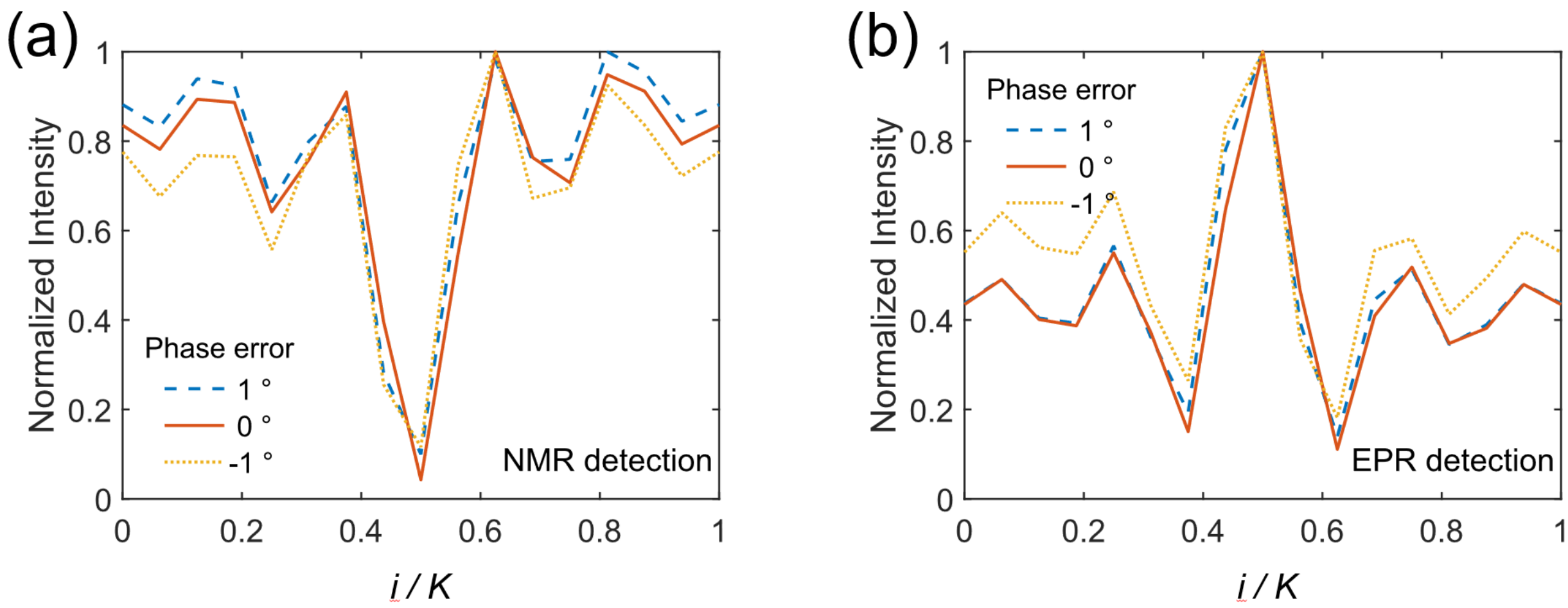

Fig. S6 Simulated influence of imperfect microwave phase control on NMR (a) and EPR (b) detected data. The phase error shown in the figures is the phase difference between the in-phase and quadrature signals from the ideal 90° in the AWG.

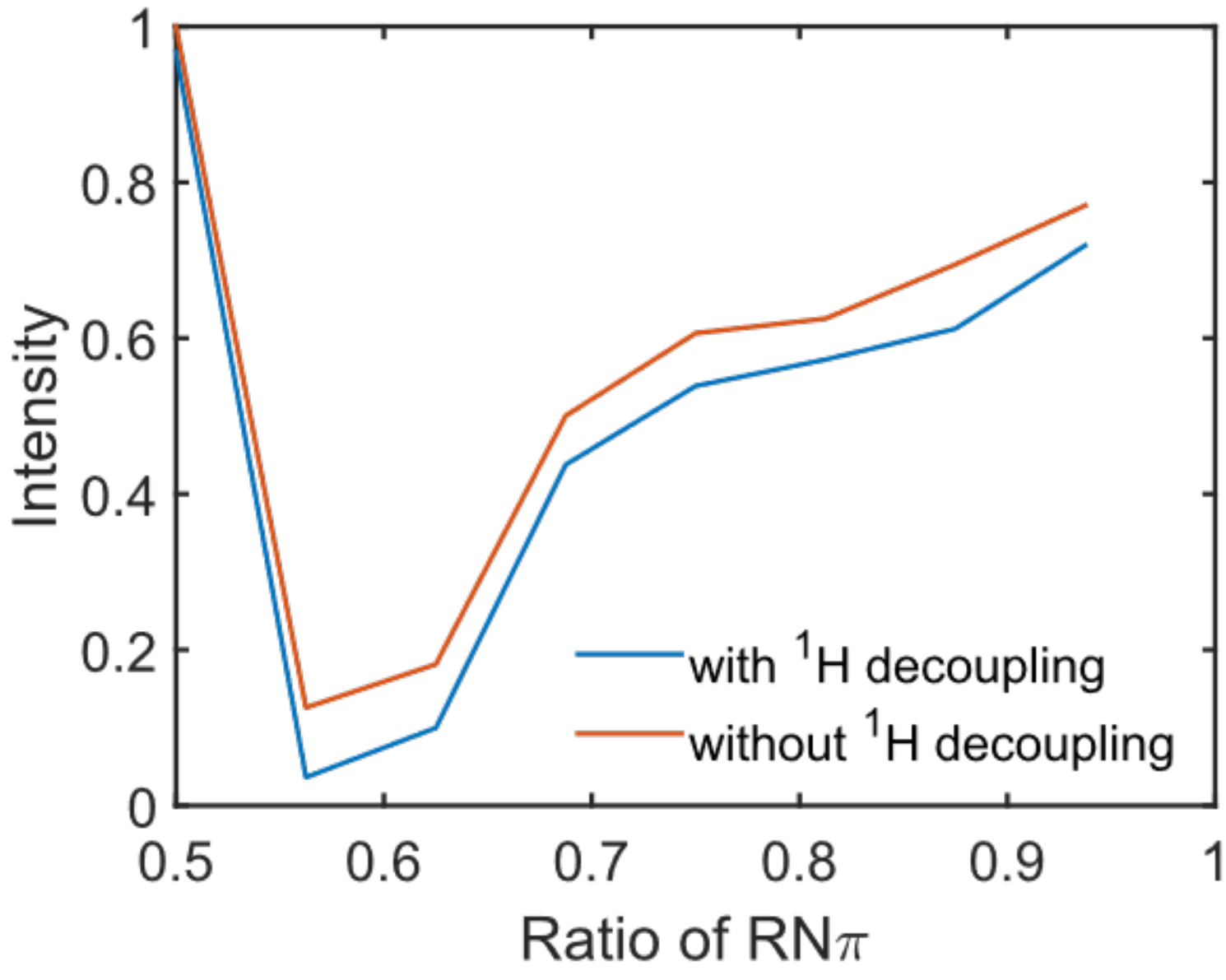

Fig. S7 Constant-time experiments with/without nuclear decoupling and EPR detection. The ^1H decoupling pulses were applied on the bulk ^1H lines with a linewidth of ~ 20 kHz, which can be sufficiently saturated or inverted with our applied rf field strength of 125 kHz.

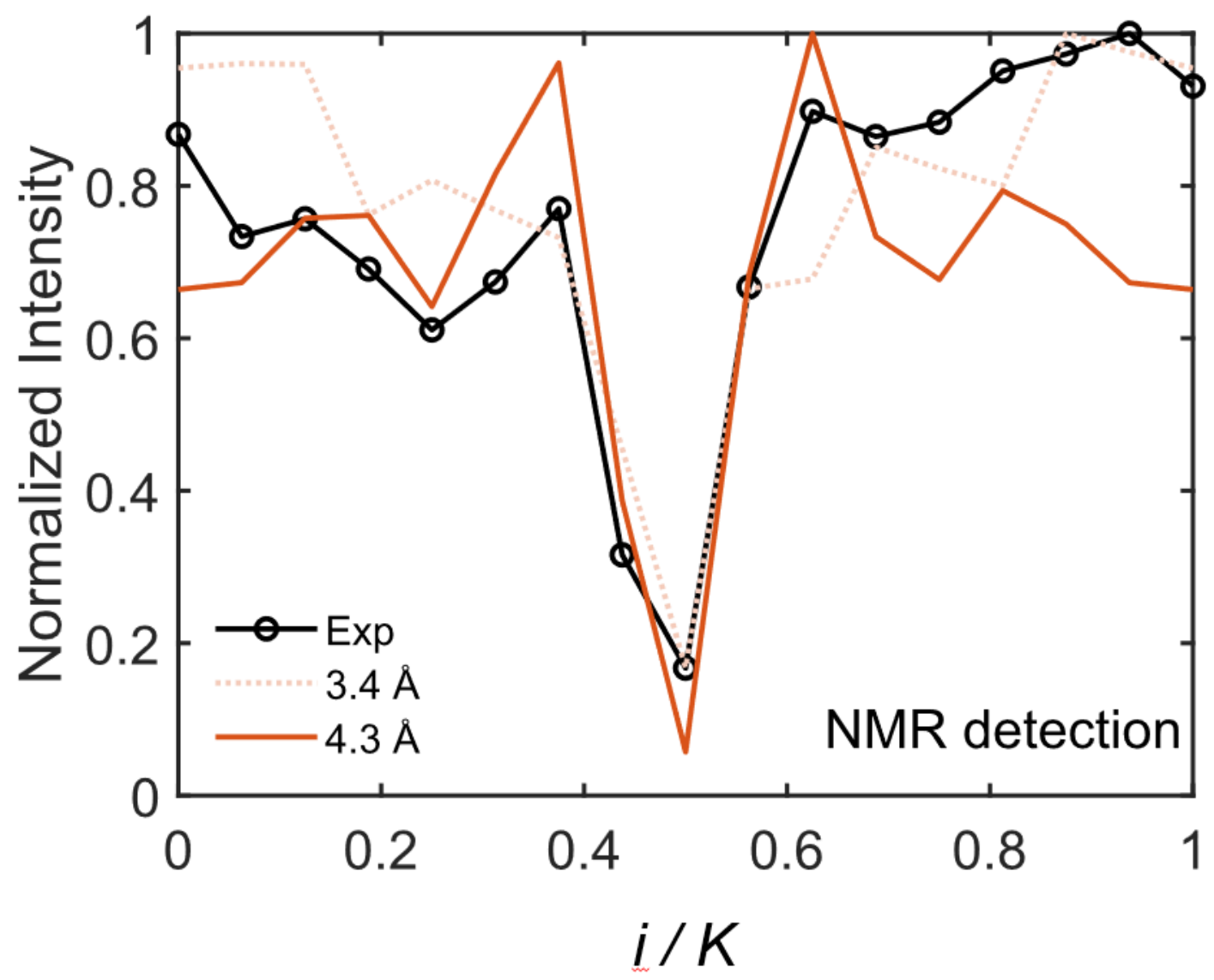

Fig. S8. Simulated curves for NMR detection in the constant time experiments using riffled $R6_4^{-1}$ with electron-nuclear distance of 3.4 and 4.3 Å.

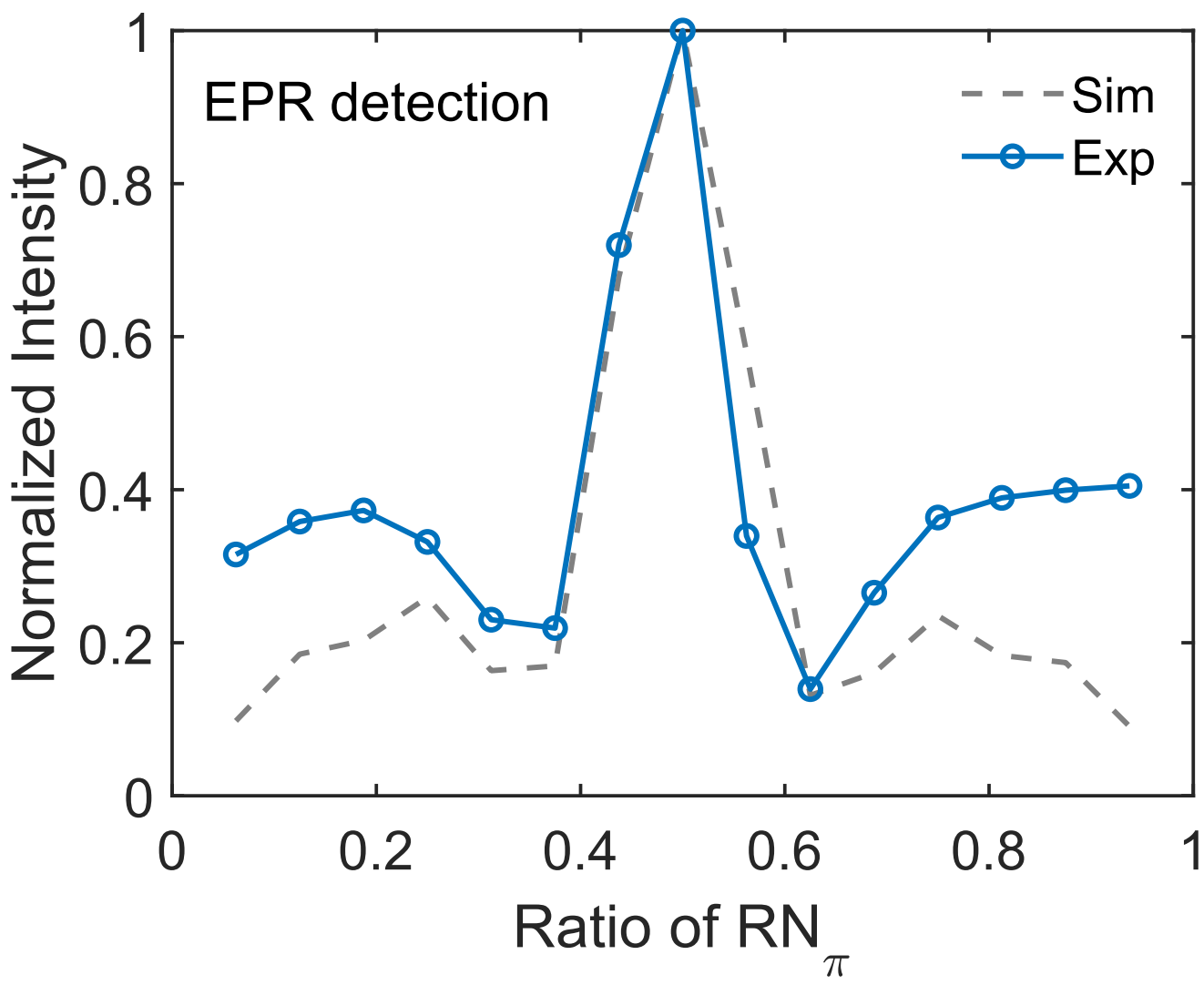

Fig. S9 Constant-time experiments using $R4_3^1$ and EPR detection. Simulations were done with an electron-nuclear distance of 4.3 Å.

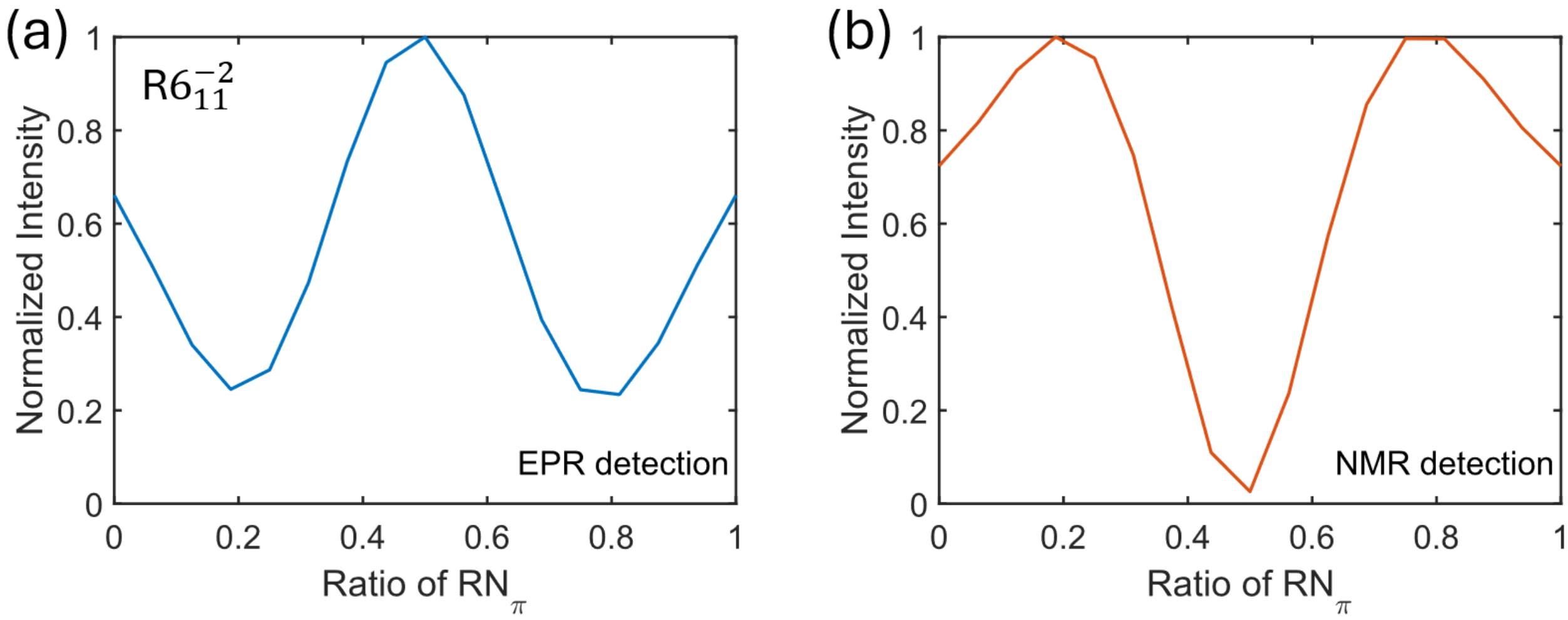

Fig. S10 Simulated constant-time experiments using $R6_{11}^{-2}$ for EPR (a) and NMR (b) detection. The basic pulse element is $90_x\, 270_{-x}$. The scaling factor of this sequence is 0.048. Although $R6_{11}^{-2}$ produces smoother curves than $R6_4^{-1}$ and $R4_3^1$, its performance is limited by the maximum traveling-wave tube (TWT) gate time (~8 μs). At 0.35 T, the proton Larmor period is approximately 68 ns. Therefore, one $R6_{11}^{-2}$ block lasts $11 \times 68\,\text{ns} = 748\,\text{ns}$. Under the 8 μs gate-time constraint, a maximum of 10 blocks can be applied, which reduces the smoothness of the resulting curve. In principle, $R6_{11}^{-2}$ would be better at higher magnetic fields. For example, moving to Q band (~1.2 T) shortens the proton Larmor period, allowing more blocks (c.a. 35) within the gate-time limit and thus showing the expected smooth behavior.

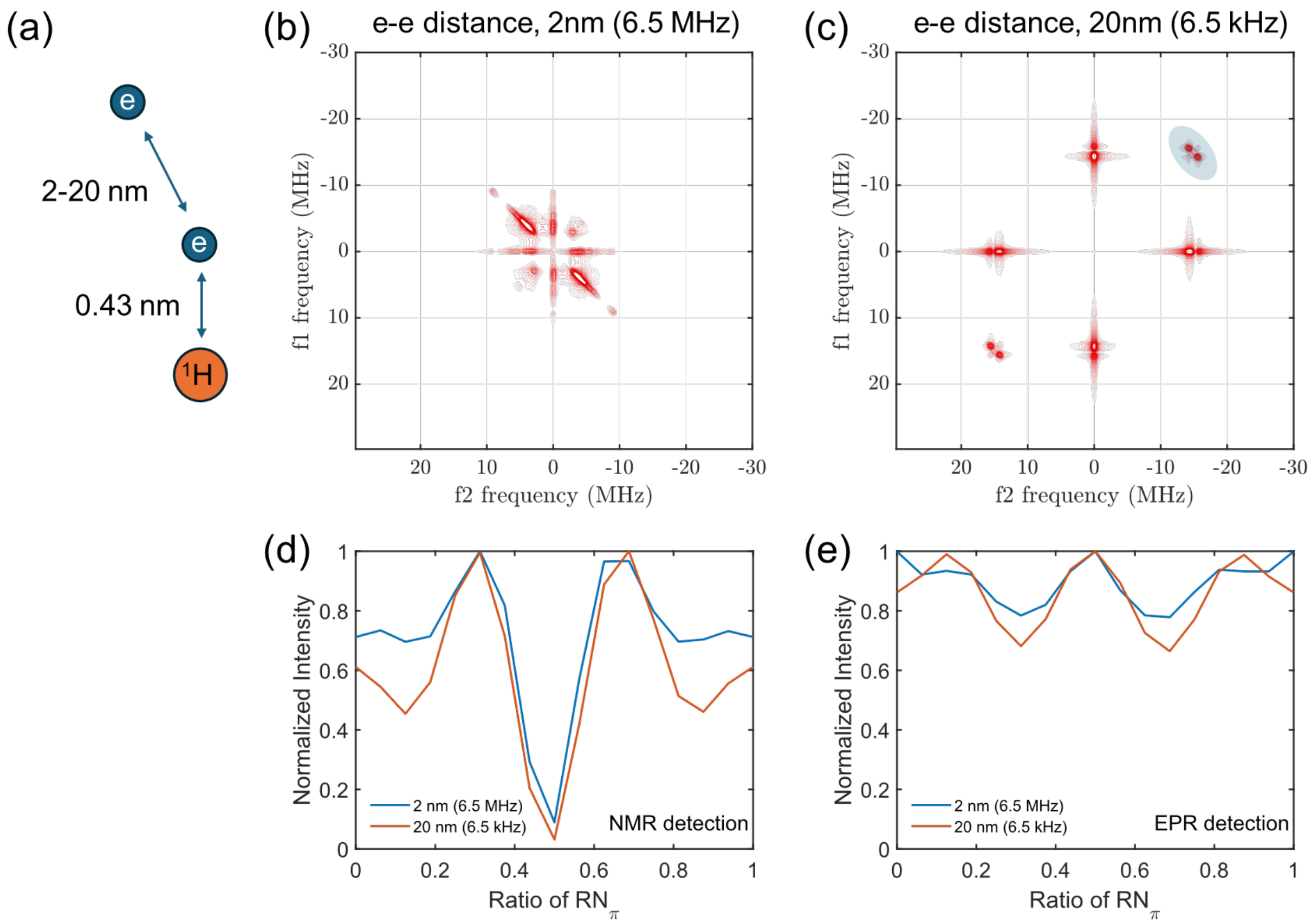

Fig. S11. Simulations examining the influence of electron–electron dipolar coupling on electron–nuclear distance measurements. (a) A three-spin model consisting of two electrons and one proton used for the simulations. (b,c) Simulated HYSCORE spectra for systems with electron–electron separations of 2 nm or 20 nm, corresponding to electron–electron dipolar couplings of 6.5 MHz and 6.5 kHz, respectively. For the 2 nm separation (6.5 MHz coupling), the electron–nuclear hyperfine features are not resolved in the HYSCORE spectrum. (d,e) Simulated polarization-transfer curves from constant-time experiments using riffled $R6_4^{-1}$ for electron–electron distances of 2 nm or 20 nm, detected via NMR (d) and EPR (e), showing largely unchanged characteristic oscillatory behaviors of the polarization-transfer curves.

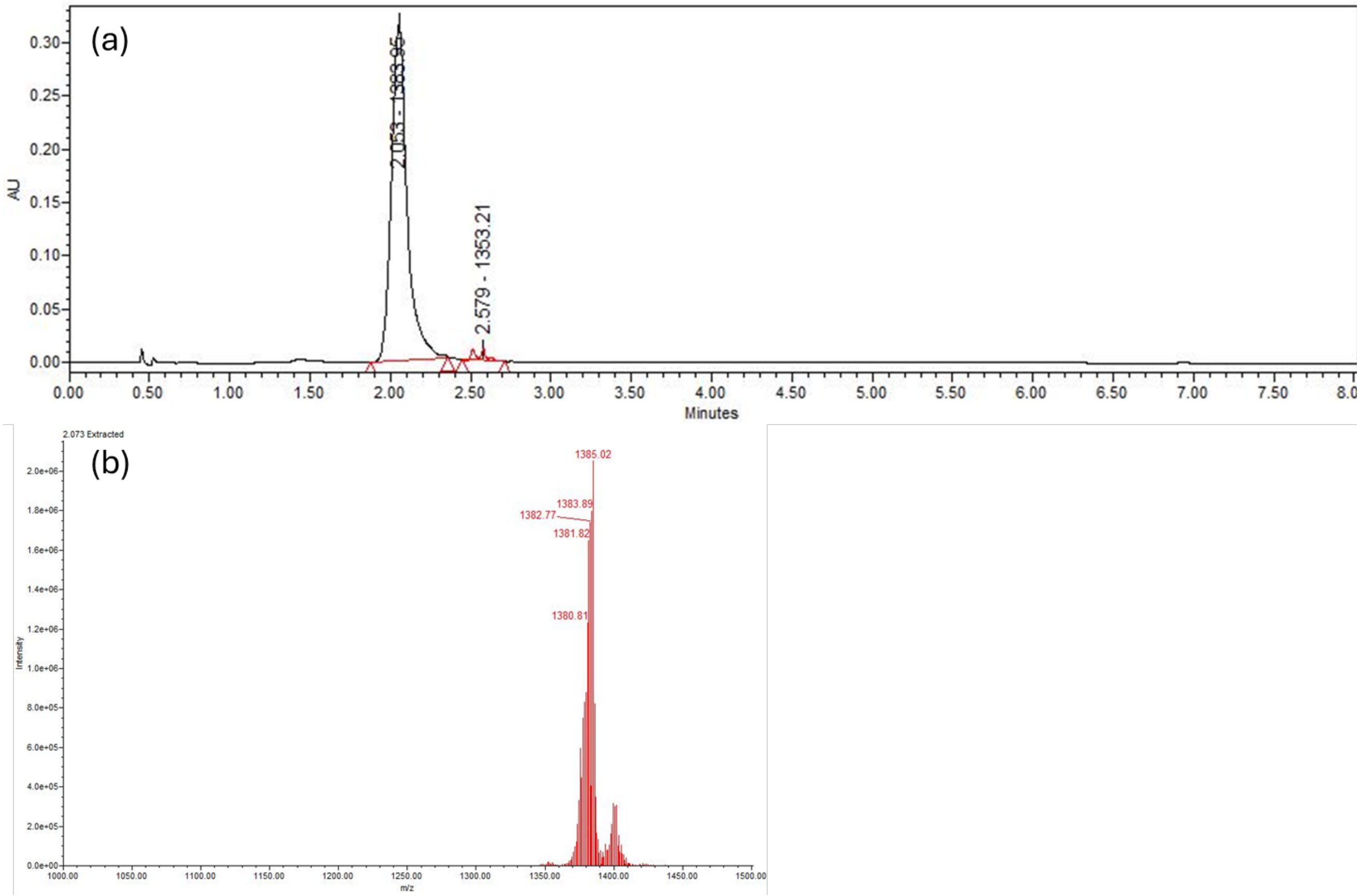

Fig. S12. HPLC-MS of Ox071 radical. (a) Chromatogram showing a purity of >97%. (b) Mass spectrum. HPLC-MS analysis was carried out using a Water Alliance e2695 separation module, a Water 2998 PDA detector, and a Water SQD2 mass detector, an XBridge BEH C18 4.6 mm x 50 mm, 2.5 μm column with a flow rate of 1.5 mL/min, a 10:1 split to the MS (0.15 mL/min). The column temperature was set to 45°C, and UV detection was from 210 to 800 nm. The m/z range was 1000-1500. The following gradient was used. 0 min: 85% H_2O, 5% ACN, 10% H_2O with 1% TFA. 1 min: 85% H_2O, 5% ACN, 10% H_2O with 1% TFA. 6 min 0% H_2O, 90% ACN, 10% H_2O with 1% TFA. 7-8 min 100% ACN.
